# Associations between depression and nature-based recreation: A cross-sectional study of adults in the United States, Spain, and Brazil

**DOI:** 10.1038/s41598-025-89156-0

**Published:** 2025-02-10

**Authors:** Claudio D. Rosa, Lincoln R. Larson, Silvia Collado, Sandra J. Geiger, Christiana C. Profice, Marcos R. T. P. Menuchi

**Affiliations:** 1 Department of Development and Environment, State University of Santa Cruz, Ilhéus, Brazil; 2 Department of Parks, Recreation and Tourism Management, North Carolina State University, Raleigh, USA; 3 Department of Psychology and Sociology, University of Zaragoza, Teruel, Spain; 4 Department of Cognition, Emotion, and Methods in Psychology, University of Vienna, Vienna, Austria; 5 Department of Health Sciences, State University of Santa Cruz, Ilhéus, Brazil; 6 Department of Physical Education, Instituto Federal do Norte de Minas Gerais, Araçuaí, Brazil

**Keywords:** Contact with nature, Depressed, Mental health, Observational study, Prevalence ratio, Human behaviour, Environmental social sciences, Psychology and behaviour

## Abstract

Cumulating evidence suggests that nature-based interventions may alleviate depression, but the association between engagement in nature-based activities and specific depressive symptoms remains unknown. We conducted a cross-sectional study to investigate how Major Depressive Disorder (MDD) symptom criteria relate to engagement in nature-based recreation (any nature-based activities, forest-based activities, gardening, nature-based adventure activities) among American (*n* = 606), Spanish (*n* = 438), and Brazilian (*n* = 448) adults (≥ 18 years old). People who reported engaging in any nature-based activities at least once per month reported experiencing all nine symptom criteria for MDD (e.g., anhedonia, feeling depressed or hopeless, sleep problems, trouble concentrating, and suicidal ideation) at lower rates than those who did not participate in nature-based recreation as frequently. Results were relatively consistent across countries and types of nature-based activities, suggesting that many forms of nature-based recreation are negatively correlated with the nine symptom criteria for MDD. The associations tended to be weaker overall among Spanish respondents. Nature-based recreation appeared to have a stronger inverse relationship with suicidal ideation than with other depressive symptoms. The cross-sectional design of this study limits the causal interpretation of the observed associations. If future experimental studies confirm our findings, practitioners across different countries can consider recommending participation in nature-based recreation to alleviate their clients’ MDD symptoms.

## Introduction

Major Depressive Disorder (MDD) is the most common depressive disorder globally, affecting almost 400 million people^1,2^. According to the fifth edition of the Diagnostic and Statistical Manual of Mental Disorders (DSM-5)^1^, to be diagnosed with MDD, a person needs to present at least five out of the nine possible symptom criteria for at least two weeks, along with clinically significant suffering or functional impairment. At least one of these five symptom criteria should be sad mood or anhedonia, and the patient’s symptomatology cannot be better explained by other causes, such as the use of substances (e.g., narcotics) or grief^1^.

The use of antidepressants and psychotherapy are two of the most well-known and recommended treatments for MDD^3,4^. Nevertheless, even the combination of these treatments normally produces only small improvements in depressive symptoms^3,4^. Thus, efforts have been directed towards complementary interventions that may help to provide greater reductions in depressive symptoms. These include physical exercise^5^, dietary changes^6^, and contact with nature^7^. The use of nature-based activities to reduce people’s depressive symptoms seems especially promising^7–9^. For instance, when compared to usual care only, participants in forest therapy groups were 17 times as likely to achieve remission and three times as likely to have at least a 50% reduction on their depressive symptoms^7^. These findings are in line with several theories and frameworks used to explain the health benefits associated with activities in nature^10–13^. Beyond the mental health benefits of positive nature experiences^10,11^, a reduction in depressive symptoms after activities in contact with nature may also occur because these activities are often associated with factors that protect against depression, such as physical exercise^5,9^ and social interactions^8,12^.

Whereas there are several ways to interact with nature, three kinds of nature-based activities have received considerable attention from researchers as strategies to improve symptoms of depression: forest-based activities^7^, nature-based adventure^9^, and horticultural activities^8^. Forest-based activities involve conducting activities in a forested area, such as walking, meditation, or nature observation, either alone or in a group.^7^. Nature-based adventure refers to any activity that contains elements of adventure (e.g., challenge, excitement) that is conducted in a natural setting^9^. Popular nature-based adventure activities are hiking, camping, mountain climbing, and surfing. Finally, horticultural activities involve planting and taking care of plants^8^, such as seeding, watering, and harvesting. Forest-based activities, nature-based adventure, and horticultural activities are usually recreational because people typically choose to do them during their free time^14^.

Despite a large body of experimental and correlational evidence supporting the potential of nature-based activities to improve adults’ depression, findings of several systematic reviews showed that no study to date has explored how these activities relate to specific symptoms of depression as assessed by a depression outcome measure^7–9^. Understanding the effect of nature-based activities on specific symptoms is relevant for practitioners (e.g., psychologists) and laypeople^15^. For instance, practitioners may decide whether nature-based activities are relevant for the specific depressive symptoms their clients are facing and laypeople may decide whether to engage or not in these activities given the symptoms they are experiencing or willing to avoid (i.e., using nature-based activities as a preventive strategy). Considering this research gap, we conducted a cross-sectional study to explore the association between nature-based recreational activities and the nine symptom criteria for MDD as registered by the Patient Health Questionnaire-9 (PHQ-9). These findings can be used to develop a hypothesis regarding the effect of nature-based activities on specific symptoms of depression, which can be tested in future experimental studies^16^.

Beyond the novelty of conducting analyses at the symptom level, our work is unique and complementary to existing evidence for several other reasons (see Supplementary Tables 1 to 3 in Supplementary File 1 for a review of the literature). First, we analyzed the association between depression and different kinds of nature-based recreational activities (i.e., forest-based activities, gardening, and nature-based adventure activities), which provides unique information about the type of nature-based activity that might be most beneficial when it comes to improving depressive symptomatology. Second, we examined whether this association varied depending on the time frame of nature engagement (e.g., last 12 months vs. typical week). Third, we explored the potential linear or “dose–response” relationship between engagement in nature-based activities and depression, which may assist in the development of hypotheses about how the frequency of nature-based activities is associated with depressive symptoms. Finally, our study constitutes one of the few studies to explore the relationship between nature-based recreation and depression separately for samples of participants from different countries (i.e., the United States of America (USA), Spain, and Brazil). This enabled us to examine whether the relationship between nature-based activities and depressive symptomatology is stable across countries.

### Methods

#### Study design and participants

We conducted a cross-sectional observational study^16^ using an online survey sampling approach. We developed the online survey using the platform Qualtrics based on the recommendations of Biffignandi and Bethlehem^17^, emphasizing an attractive online survey layout and the need for brevity. No incentives were offered to take part in this online survey. We collected IP addresses and emails. Duplicate IP addresses were possible because people from the same household/classroom could reply to the questionnaire. We observed some duplicate IP addresses, but responses were linked to different email addresses.

The study was approved by the ethics committee at the Universidade Estadual de Santa Cruz (CAAE: 56,750,322.0.0000.5526). Participants consented to have their information used for research purposes, including sharing of fully anonymized data. All methods were performed in accordance with the relevant guidelines and regulations^16,18^. The English version of the questionnaire was distributed to a random sample of 5,000 undergraduate and 2,500 graduated student emails from North Carolina State University (NCSU), USA. As we did not have access to a random sample of students’ emails in Spain and Brazil, emails were sent with an invitation letter to the contact list of the Spanish and Brazilian authors of this manuscript, and through the social media, such as WhatsApp, Facebook, and Instagram. These countries were selected for convenience. In the invitation letter, we invited people to participate in our survey and to share it with their contacts. We tried to reach as many participants as possible to get narrower confidence intervals (CIs) for our estimates, so no formal sample size calculation was performed^19^. Most participants spent less than 10 min voluntarily completing our online survey (the median time to complete the survey was 6.7 min). All data were collected between February 01 and June 26, 2023.

People aged ≥ 18 years who were able to answer the questionnaire were eligible for this study. For the USA sample, participants had to additionally be university students at the NCSU. In total, 1,853 people clicked on the link to start this online survey. Nonetheless, some participants were eliminated because they were younger than 18 years old (USA: *n* = 1; Spain: *n* = 2; Brazil: *n* = 2) or did not report their age. We used the available data for the participants who completed at least 96% of the survey because these participants only failed to proceed to the last page to send the completed survey. In total, 1,492 valid responses remained, which corresponds to a completion rate of 80.5% of all people who clicked on the survey link (Table 1).

**Table 1:** Participants’ sociodemographic characteristics.

Variable %	Survey version		
	English	Spanish	Portuguese-Brazil
Number of valid responses	606	438	448
Age	Mean = 23.7 Range = 18 to 61	Mean = 26.4 Range = 18 to 83	Mean = 32.7 Range = 18 to 70
18 to 39	96.7	89.0	77.0
≥ 40	3.3	11.0	23.0
Gender	-	-	-
Man	42.6	24.7	32.8
Women	54.5	73.7	66.1
Gender variant/Non-confirming	3.0	0.7	1.1
Not listed	0.0	0.9	0.0
Highest education	-	-	-
Secondary (High) school	50.8	32.8	30.4
Vocational school	1.8	11.2	3.8
College or University	27.6	34.9	33.9
Graduate (Master or equivalent)	18.6	14.4	22.1
Doctoral Degree	1.2	6.7	9.8
Family income	-	-	-
Well below average	9.6	4.1	5.8
Slightly below average	16.0	14.4	15.8
Average	30.9	57.8	48.2
Slightly above average	32.8	21.8	23.0
Well above average	10.7	1.8	7.1
Urbanicity	-	-	-
Urban	75.9	64.8	90,2
Rural	17.5	27.2	4,5
Not sure	6.6	8.0	5,4
Skin color	-	-	-
Whiter (2 or less in the NIS Skin Color Scale)	74.8	80.3	35.9
Darker (3 or more in the NIS Skin Color Scale)	25.2	19.7	64.1
Country participants were living			
United States of America	99.7	0.2	0.4
Spain	0.0	98.9	0.0
Brazil	0.0	0.0	98.2
Other	0.3^a^	0.9^b^	1.3^c^

^a^Two participants were living in India when they filled in the English survey. ^b^Two participants were living in Italy, one in Madagascar, and one in France when they filled in to the Spanish survey. ^c^Two participants were living in Peru, one in France, one in Portugal, and one in the Equator when they filled in the Portuguese-Brazil survey.

The percentage of missing data was below 1% on any variable. In all three survey versions, the average participant was a young adult (mean age 27.2 years). Most participants were women, well educated, held an average or close to average family income compared to other people living in their country, and lived in urban areas. Most participants in the English and Spanish surveys reported they had a white or close to white skin color, and most Brazilians reported having a dark or close to dark skin color. Few participants in the English (*n* = 2), Spanish (*n* = 4), and Brazilian (*n* = 5) surveys were not living in the USA, Spain, or Brazil at the moment of data collection (Table 1).

### Measures

We gathered information about participants’ contact with nature, depressive symptoms, and sociodemographic data. The online survey was created in English and translated into Spanish and Portuguese. After translating the survey to Spanish and Portuguese, we translated it back to English. Then, we compared the original and translated English versions. After resolving minor translation issues, we conducted interviews with potential participants, and no problems with item comprehensibility were identified^20^.

#### Contact with nature

We developed items to assess participants’ frequency of participation in nature-based recreation based on previous studies e.g.,^21,22^. This was done because, to date, there is no validated instrument to assess participation in specific nature-based recreational activities. Then, we asked three experts with experience studying the effect of contact with nature on human health to give their opinion regarding the first draft of our items, with special attention to the items’ comprehensibility. Based on the experts’ feedback, we edited the content of the items until we felt that all items were comprehensible and the response options were appropriate. The items included a general question about the frequency of participation in any nature-based recreation plus one item for each of three types of activities: Forest-based activities, gardening, and nature-based adventure^23,24^. Before replying to the questions, participants read the definition of nature-based recreation: “Nature-based recreation and leisure activities are activities in contact with nature that you choose to do during your free time”. For each type of nature-based recreation, we assessed both participation in the past 12 months and participation during a typical week. The question covering participation in any nature-based recreational activity in the past 12 months was the following: “In the past 12 months, which of the following best describes your participation in ANY type of nature-based recreation and leisure activities?”. Response options were: I never participated; I rarely participated (a few times a year); I sometimes participated (about once a month); I often participated (several times each month); I very often participated (pretty much every week). The engagement in any activity during a typical week was assessed with the following question “On how many days in a typical week do you participate in ANY type of nature-based recreation and leisure activities? [For example, if you typically go to the beach 2 days in a week and garden on 2 different days out of that same week, your answer would be 4 days.]”. Response options were: None (never participate); 1 day; 2 days; 3 days; 4 days; 5 days; 6 days; Every day. The complete surveys in English (https://univiepsy.qualtrics.com/jfe/form/SV_7V5aUWIZsWwEsbY), Spanish (https://univiepsy.qualtrics.com/jfe/form/SV_0pIe471jMeI2WoK), and Portuguese-Brazil (https://univiepsy.qualtrics.com/jfe/form/SV_ac68XwULUWx3L5I) are available online. The complete questionnaire is available on Supplementary File 1.

#### Depressive symptoms

The PHQ-9 was used to assess the nine symptom criteria used to diagnose MDD^25^. This scale was translated to several languages by Pfizer using the translation approach recommended by the World Health Organization (WHO). This includes Spanish^26^ and Portuguese-Brazil versions^27^. These versions have been used by many previous studies and their coverage of the MDD diagnostic criteria praised^27,28^. Previous studies have shown that the PHQ-9 mean score remains stable over time without any intervention^25^ (indicating high test–retest reliability), and a PHQ-9 score ≥ 10 was established as the optimum criterion to screen for MDD in a diversity of settings^29^. We used this cutoff because it is associated with the best combination of sensitivity and specificity in the identification of MDD^29^. It should be noted that the PHQ-9 is a screening tool, so scoring ≥ 10 would not necessarily lead to a MDD diagnosis.

#### Sociodemographic information

We collected information about participants’ age, gender, highest educational level, average family income, skin color, urbanicity, and country (see Table 1). Information about skin color was collected using the New Immigrant Survey (NIS)^30^. No cutoffs were provided by the NIS authors, so we selected the cutpoint that, in our view, best differentiates a white from a non-white skin tone. We also collected information about participants’ ethnicity, but we deemed this less comparable across countries than skin color. Thus, ethnicity was not used in this analysis.

### Data analyses

We used descriptive statistics to describe the overall frequency of nature-based recreation, depressive symptoms, and sociodemographic information in our samples. We then assessed relationships between the frequency of nature-based recreation and PHQ-9 scores using means as well as a dichotomized approach to facilitate the interpretation of results^16^. We dichotomized the frequency of nature-based recreation engagement during the past 12 months into less than monthly (code = 0) and at least monthly (code = 1). We chose this timeframe because engaging in nature-based recreational activities once per month may be an achievable goal for most people^21,22^. A similar logic was followed to dichotomize the frequency of engagement in nature-based recreation during a typical week. We used code 0 for participants who reported not engaging in nature-based recreational activities at all, and 1 for participants who reported engaging in these activities at least one day during a typical week. Moreover, we used code 0 for participants with scores lower than 10 in the PHQ-9 and 1 for participants with a score ≥ 10 on this scale because a PHQ-9 score of ten or greater is the optimum screening criterion for MDD^29^. Finally, we used code 0 for participants who reported being “Not at all” bothered by specific depressive symptoms (e.g., anhedonia) and 1 for participants who reported being bothered at least “Several days” during the last two weeks.

We used fixed-effects meta-analyses^31^ to estimate the mean difference in PHQ-9 scores between groups dichotomized according to the frequency of participation in nature-based recreation (as described above). When working with mean differences, we report results in a standardized metric (Hedges’*g*) beyond the raw mean differences. Similarly, we estimated the Prevalence Ratio (PR) of a PHQ-9 score ≥ 10 between the different nature-based recreation groups^32^. Prevalence Ratios (PRs) are calculated using the same formulae than Risk Ratios (RRs). The former term is used in cross-sectional studies and the later in the longitudinal ones. The term “Risk Ratio” appears in the forest plots in Supplementary File 1 because this is the default term used by the statistical software RevMan. We also calculated PRs for the nine specific symptom criteria assessed by the PHQ-9, comparing nature-based recreational groups. All inferential analyses were conducted using the free software RevMan^33^. We opted for PRs because they are easier to understand than odds ratios, and we ran fixed-effects (instead of random effects) meta-analyses because estimates of statistical heterogeneity are imprecise with very few studies^31,34^. Lower overlap among CIs of effect sizes is associated with higher statistical heterogeneity, and CIs that include the value of 0 for mean differences or of 1 for PRs suggest non-statistically significant results^34^. We considered each sample (i.e., Americans, Spanish, and Brazilians) as a different study^34^. Meta-analysis was used to facilitate easy and clear visualization of each sample estimate through a forest plot and to provide a (weighted) combined estimate from the three samples^34^. As a sensitivity analysis, we assessed the probability of having a PHQ-9 score ≥ 10 depending on the frequency of participation in any nature-based recreational activity using different approaches to categorize the latter variable. This helps to understand how different choices related to the dichotomization of our nature-based recreation variables would influence our results. These analyses are also informative regarding a possible “dose–response” relationship between participation in nature-based recreational activities and depression. Finally, as another type of sensitivity analysis, we examined whether our results were consistent regardless of participants’ gender, family income, and skin color. The data sheets with all inferential analyses are shared as Supplementary Files 2 and 3.

## Results

A small portion (6.4%) of participants reported that they had never engaged in any nature-based recreation in the past 12 months, and 74.7% reported they had participated in nature-based recreational activities at least once a week during a typical week. Among the activities evaluated in this study, the most practiced were forest-based activities, followed by nature-based adventure, and gardening. Concerning depression, Brazilians presented the highest scores in the PHQ-9, followed by the North Americans and Spanish. In terms of the PHQ-9 cutoff, 31.8% of Americans, 25.2% of Spanish, and 53.6% of Brazilians held a PHQ-9 score ≥ 10. Some depressive symptoms were highly prevalent in the sample. For example, 87.5% of the participants reported feeling tired or having little energy for at least several days over the last two weeks. Detailed descriptive information related to participants’ nature-based recreation and depressive symptoms are available in Supplementary File 1 (see Supplementary Tables 4 to 9).

### Relationship between nature-based recreation and depressive symptoms

We assessed the difference in PHQ-9 mean scores according to whether participants engaged (at least monthly, during the last 12 months) in nature-based recreational activities or did not (Table 2). Across all samples and activities, the average PHQ-9 score of participants who engaged at least monthly in nature-based recreation was lower than the ones who did not engage that frequently. This mean difference in PHQ-9 scores was close to or greater than 1.5 points for all activities. Hedges’ *g* for the annual timeframe ranged from 0.28 for gardening to 0.44 for any nature-based recreational activity. The mean differences tended to be smaller in the Spanish sample and larger in the Brazilian and English samples, producing high statistical heterogeneity in the meta-analyses (i.e., little overlap among the CIs of the estimates, see Supplementary Fig. 1 in Supplementary File 1). The results were similar when considering participation in nature-based recreational activities on a typical week, indicating that participants who engaged at least weekly in nature-based recreation during a typical week held lower mean PHQ-9 scores than the ones who did not engage in nature-based activities that frequently (Table 2, and also Supplementary Fig. 2 in Supplementary File 1). Mean differences ranged from 0.92 for gardening to 2.41 for any nature-based recreational activity (Table 2). Hedges’*g* for the typical week timeframe ranged from 0.18 for gardening to 0.45 for any nature-based recreational activity. Among Americans, the association between gardening and depression mean scores tended to be weaker than the association between the other nature-based activities and depression mean scores. This was consistent in both timeframes (past 12 months and typical week). Also, in both timeframes, the 95% CI of estimates covered zero for all the nature-based activities in the Spanish sample, and for gardening among Americans (Supplementary Figs. 1 and 2 in Supplementary File 1).

**Table 2:** Fixed-effects meta-analyses of the mean differences in PHQ-9 scores (95% CI) according to participation in nature-based recreation. Estimates are based on pooled data from the North American, Spanish, and Brazilian Samples.

	At least Monthly			At least Weekly		
	Mean difference^a^	Lower boundary of the 95%CI	Higher boundary of the 95%CI	Mean difference	Lower boundary of the 95%CI	Higher boundary of the 95%CI
Any nature-based recreation activity	2.26	1.62	2.89	2.41	1.70	3.12
Forest-based	2.14	1.54	2.74	1.56	0.94	2.18
Gardening	1.46	0.86	2.06	0.92	0.33	1.50
Nature-based adventure	1.90	1.32	2.48	1.64	1.07	2.21

^a^Positive mean differences indicate that participants who engaged in nature-based recreation at least monthly during the past 12 months or weekly during a typical week held LOWER PHQ-9 scores than participants who did not.

Still at the PHQ-9 score level, we assessed the number of participants with a PHQ-9 score ≥ 10 according to whether participants engaged at least monthly in nature-based recreation in the last 12 months. Participants who were not engaged at least monthly in any nature-based recreational activity had a 73% higher prevalence of a PHQ-9 score ≥ 10 than those who did engage in nature-based recreational activities at least monthly (Table 3). A higher prevalence was also observed for forest-based activities, gardening, and nature-based adventure activities. The PR tended to be similar across countries. As a result, statistical heterogeneity was zero or close to zero in most analyses (Supplementary Fig. 3 in Supplementary File 1). The patterns were again similar for the questions regarding engagement in nature-based activities during a typical week (Table 3, and also Supplementary Fig. 4 in Supplementary File 1). For example, participants who did not engage at least weekly in any nature-based recreational activity had a 70% greater prevalence of a PHQ-9 score ≥ 10 than those who did engage in nature-based recreational activities at least weekly. Similar to mean depression scores, the association between the prevalence of a PHQ-9 score ≥ 10 and gardening was weaker than the association between the prevalence of these high PHQ-9 scores and the other nature-based recreational activities evaluated, among Americans. In both timeframes, 95% CIs covered relatively strong associations as well as no association between gardening and the PHQ-9 cutoff among Americans. Similarly, some of the associations between nature-based recreation (e.g., gardening) and the PHQ-9 cutoff were not statistically significant for the Spanish sample (Supplementary Figs. 3 and 4 in Supplementary File 1).

**Table 3:** Fixed-effects Meta-analyses Estimating the Prevalence Ratio of Holding a PHQ-9 Score ≥ 10 According to Participation in Nature-Based Recreation. Estimates are Based on Pooled Data from the North American, Spanish, and Brazilian Samples.

	At least monthly			At least weekly		
	Prevalence Ratio	Lower boundary of the 95%CI	Higher boundary of the 95%CI	Prevalence Ratio	Lower boundary of the 95%CI	Higher boundary of the 95%CI
Any nature-based recreation activity	1.73	1.51	1.97	1.70	1.49	1.93
Forest-Based	1.67	1.44	1.94	1.53	1.33	1.75
Gardening	1.51	1.28	1.79	1.26	1.09	1.45
Nature-based adventure	1.68	1.41	1.99	1.67	1.43	1.95

A Prevalence Ratio greater than 1 indicates that participants who were not engaged at least monthly during the last 12 months or weekly during a typical week in nature-based recreation activities were MORE likely to hold a PHQ-9 score ≥ *10* than the participants who did engage in those activities more frequently.

Regarding the results at a symptom level, we assessed the likelihood of participants having been bothered by each of the nine PHQ-9 symptom criteria over the last two weeks according to the frequency in which they had engaged in nature-based recreational activities (Table 4). Compared to participants who engaged in nature-based recreational activities at least monthly, those who not were engaged in nature-based recreational activities as frequently were more likely to have been bothered by all nine depressive symptom criteria registered by the PHQ-9. These results were similar for each specific nature-based activity (i.e., forest-based activity, gardening, and nature-based adventure activity). Again, similar results were found when the frequency of engagement in nature-based recreational activities during a typical week was considered. The Spanish sample generally had smaller differences in prevalence, producing considerable statistical heterogeneity in many analyses (see Supplementary File 2). The relationship between gardening and some depressive symptoms (e.g., trouble concentrating) was weaker and sometimes in line with a lack of association (Table 4).

**Table 4:** Results from Fixed-Effects Meta-Analyses Estimating the Prevalence Ratio [95% CI] of being Bothered by Each of the Nine Symptom Criteria assessed by the PHQ-9. Estimates are based on pooled data from the North American, Spanish, and Brazilian Samples.

	Any nature-based recreational activity		Forest-based		Gardening		Nature-based adventure	
	Monthly	Weekly	Monthly	Weekly	Monthly	Weekly	Monthly	Weekly
Little interest or pleasure in doing things	1.13 [1.06, 1.20]	1.06 [1.00, 1.13]	1.13 [1.06, 1.20]	1.10 [1.04, 1.17]	1.11 [1.03, 1.19]	1.04 [0.98, 1.10]	1.11 [1.04, 1.19]	1.10 [1.04, 1.18]
Feeling down depressed or hopeless	1.22 [1.13, 1.31]	1.22 [1.13, 1.31]	1.22 [1.12, 1.32]	1.15 [1.07, 1.24]	1.14 [1.05, 1.25]	1.10 [1.02, 1.20]	1.24 [1.13, 1.35]	1.17 [1.08, 1.27]
Trouble falling or staying asleep or sleeping too much	1.10 [1.03, 1.17]	1.07 [1.00, 1.15]	1.14 [1.06, 1.22]	1.06 [0.98, 1.13]	1.08 [1.00, 1.17]	1.02 [0.95, 1.09]	1.14 [1.06, 1.23]	1.11 [1.04, 1.19]
Feeling tired or having little energy	1.06 [1.02, 1.10]	1.04 [1.00, 1.09]	1.06 [1.01, 1.10]	1.05 [1.01, 1.09]	1.09 [1.04, 1.14]	1.07 [1.03, 1.12]	1.07 [1.02, 1.12]	1.06 [1.02, 1.11]
Poor appetite or overeating	1.15 [1.06, 1.25]	1.12 [1.03, 1.22]	1.16 [1.07, 1.26]	1.11 [1.02, 1.20]	1.10 [1.00, 1.20]	1.06 [0.98, 1.15]	1.25 [1.14, 1.37]	1.14 [1.05, 1.24]
Feeling bad about yourself or that you are a failure	1.20 [1.09, 1.32]	1.12 [1.02, 1.23]	1.27 [1.15, 1.40]	1.12 [1.02, 1.23]	1.10 [1.00, 1.22]	1.01 [0.92, 1.11]	1.21 [1.10, 1.34]	1.14 [1.04, 1.26]
Trouble concentrating	1.14 [1.05, 1.22]	1.12 [1.03, 1.21]	1.11 [1.03, 1.20]	1.05 [0.97, 1.14]	1.15 [1.03, 1.28]	1.01 [0.93, 1.09]	1.12 [1.03, 1.22]	1.09 [1.01, 1.18]
Agitation or retardation	1.24 [1.09, 1.41]	1.19 [1.04, 1.37]	1.21 [1.05, 1.40]	1.11 [0.97, 1.27]	1.10 [0.95, 1.27]	1.02 [0.90, 1.17]	1.30 [1.12, 1.51]	1.09 [0.95, 1.25]
Thoughts that you would be better off dead or of hurting yourself	2.11 [1.66, 2.68]	1.77 [1.39, 2.25]	1.81 [1.39, 2.35]	1.34 [1.06, 1.70]	1.48 [1.12, 1.96]	1.45 [1.13, 1.87]	1.52 [1.16, 1.99]	1.40 [1.09, 1.78]

A Prevalence Ratio greater than 1 indicates that participants who were not engaged in nature-based recreational activities at least monthly during the last 12 months or weekly during a typical week were MORE likely to be bothered by a depressive symptom than the participants who did engage in nature-based recreational activities more frequently.

### Sensitivity analysis and potential “dose–response” relationship between nature-based recreation and depression

**Fig. 1:**
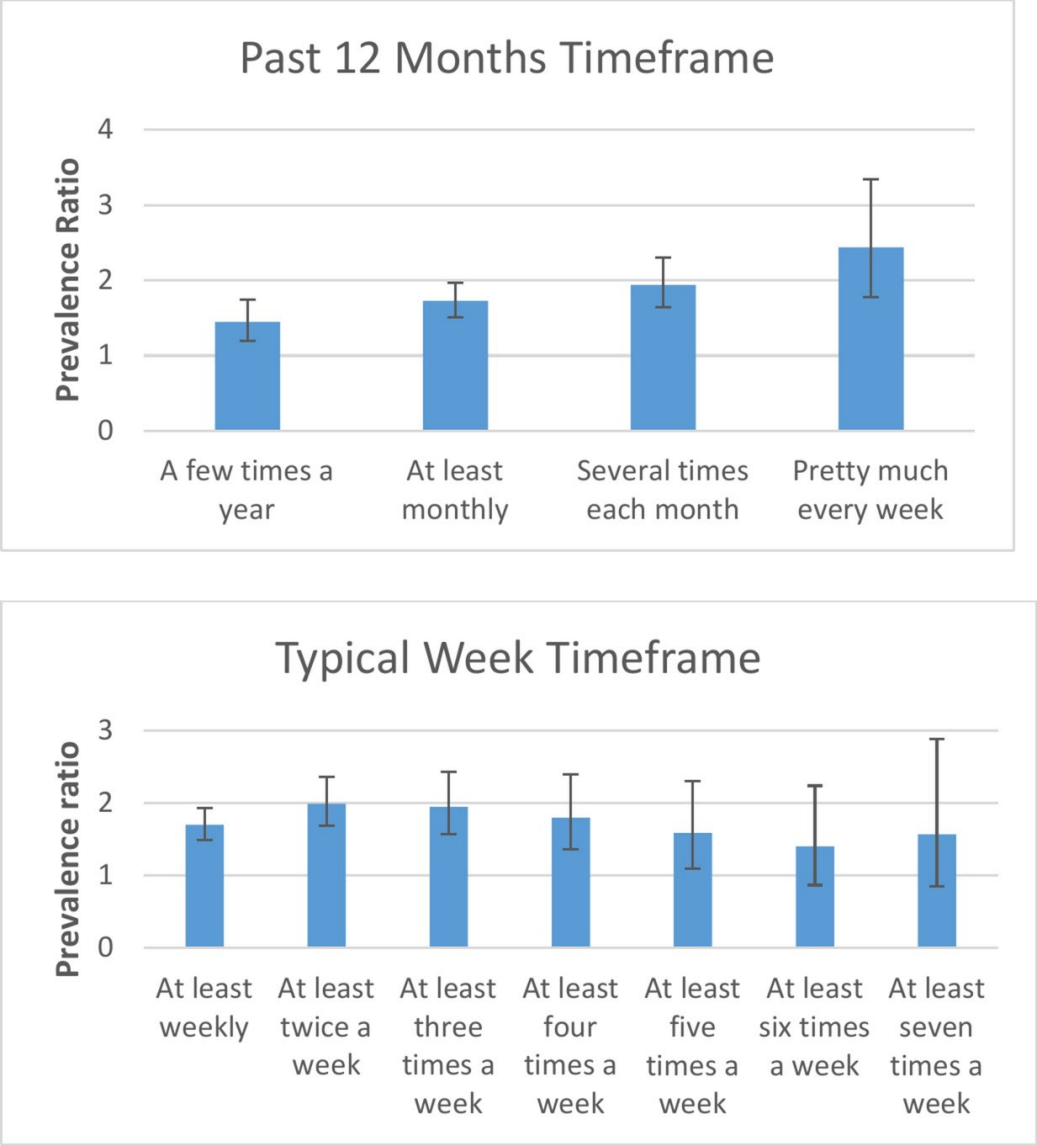
Fixed-effects Meta-Analyses Estimating the Prevalence Ratio and 95% CI of Holding a PHQ-9 Score ≥ 10 According to Binary Categorizations of Participation in Different Levels of Nature-Based Recreation, Relative to Lower Levels of Recreation. Prevalence Ratios are Based on Pooled Data from the North American, Spanish, and Brazilian Samples. A Prevalence Ratio greater than 1 indicates that participants who were not engaged during the last 12 months or during a typical week in any nature-based recreational activities were MORE likely to hold a PHQ-9 score ≥ 10 than the participants who did engage in nature-based activities that often. Greater Prevalence Ratios represent stronger associations than smaller Prevalence Ratios.

As a sensitivity and “dose–response” analysis, we assessed the probability of having a PHQ-9 score ≥ 10 depending on the frequency of participation in any nature-based recreational activity using different approaches to dichotomize the latter variable (Fig. 1). Considering the past 12 months as a timeframe, the highest PR was obtained when individuals who had engaged in any nature-based recreational activities pretty much every week (the response option that indicates the highest frequency of engagement) were compared with individuals who had not engaged in these activities that frequently (PR = 2.44). Lower PRs were observed for the categories that involve less frequent engagement in nature-based activities, in a linear fashion (Fig. 1). This “dose–response” pattern was not observed in the typical week timeframe. For example, individuals who had not engaged at least seven times a week in any nature-based recreational activity had a 57% higher prevalence of PHQ-9 scores ≥ 10 than individuals who engaged in any nature-based recreational activity that frequently. The difference in prevalence was higher when individuals who had not engaged at least twice a week were compared with individuals who engaged that frequently in any nature-based recreational activity (PR = 1.99). The negative correlation between engagement in any nature-based recreational activity and having a PHQ-9 score ≥ 10 remained across the many different dichotomization strategies of the nature-based recreation variables that we tested (Fig. 1).

Finally, we checked whether our results were consistent across participants’ gender, family income, and skin color (Supplementary Table 10 in Supplementary File 1). We found, independent of participants’ gender (man or woman), family income (up to average or more than average) and skin color (whiter or darker), a higher prevalence of a PHQ-9 ≥ 10 scores in participants who had not engaged in any nature-based recreational activity at least monthly (during the last 12 months) than in participants who engaged that frequently.

## Discussion

We examined the relationship between nature-based recreation and the MDD symptom criteria among people in three different countries (Americans, Spanish, and Brazilians) and found a consistent pattern across all samples: Nature-based recreation was negatively correlated with these nine symptom criteria registered by the PHQ-9. These results extend previous findings since no previous study assessed the association between specific depressive symptoms and depression as assessed by a depression outcome measure (see Supplementary File 1 for a literature review). Results were relatively consistent across the different types of nature-based activities considered (i.e., any nature-based recreational activity, forest-based activities, gardening, or nature-based adventure activities). Together with previous randomized trials^35–41^, these results suggest that benefits from nature-based recreation may be achieved through a diversity of activities. Nonetheless, compared to other nature-based activities, the associations between gardening and depressive symptoms tended to be weaker for Americans. It might be that gardening practices among American university students diverged from the practices of Spanish and Brazilian participants in our study, as the latter samples were not limited to students. Moreover, similar to previous studies^42^, we observed weaker associations between nature-based recreation and health among Spanish than among people from other countries. In fact, the 95% CIs of some of these weaker associations were also compatible with an absence of association in the population^19,43^. It is possible that Spanish people experience a different relationship between nature-based recreation and mental health than Americans and Brazilians. It might be that a greater proportion of Spanish participants than Americans and Brazilians engage in nature-based recreation to alleviate some existing health problem (e.g., anxiety or depression), making the differences between people who engage in these activities and the ones who do not engage less accentuated^44^. Given these weaker and sometimes non-statistically significant associations, especially in Spain but also for gardening in America, larger studies are needed to support our findings.

The relationship between engagement in nature-based recreational activities and depressive symptoms was also consistent across the different time frames considered in this study (i.e., in the past 12 months or a typical week), suggesting that an achievable frequency of contact with nature (e.g., monthly or weekly) is associated with reduced depressive symptoms. However, the negative correlation between contact with nature and depression is not necessarily linear (Fig. 1). One possible explanation for our results is that there is a reduction in the prevalence of depressive symptoms when participation in nature-based recreation increases up to a twice-a-week basis, but a higher frequency of participation in nature-based recreational activities might not provide additional benefits (Fig. 1). From a more conservative perspective, our results indicate that there may not be large differences in the prevalence of depressive symptoms between people who engage in nature-based recreation two days a week and those who engage three days a week. These findings are consistent with previous research examining this relationship (see Supplementary Table 3 in Supplementary File 1 for a summary of previous studies that examined the “dose–response” relationship between nature-based activities and depressive symptoms), as well as other studies suggesting that even small doses of nature may be beneficial for mental health^45^.

Our study was the first to explore associations between nature-based recreation and the nine symptom criteria for MDD assessed by the PHQ-9. We found that people who engaged in nature-based recreation at least monthly suffered less from all of these symptom criteria compared to the ones who were not engaged that frequently. The results were relatively similar for the typical week timeframe – people who engaged in nature-based recreation at least once a week were less likely to report any of these symptom criteria. The differences in prevalence were relatively small for most symptoms but were considerably larger for suicidal ideation (Table 4). This may be because suicidal ideation is influenced by the other eight symptom criteria^46^. For instance, a person who has sleep problems and anhedonia may be more likely to think about hurting herself/himself than a person who does not have any of these symptoms^47^. Thus, participation in nature-based recreation may substantially reduce the probability of suicidal ideation by improving other depressive symptoms. It is worth mentioning that, in some cases, the estimates of the association between nature-based recreation and depressive symptoms were not statistically significant (i.e., in our sample, the estimates suggest an association, but as the CIs overlap 1 no association is also plausible in the population), such as for the symptom criterion ‘Agitation and Retardation’ and several estimates for gardening. Thus, larger studies are needed to confirm the observed associations.

Our findings are in line with many theories and frameworks that explain the health benefits of engaging in activities in contact with nature^10–12^ and with previous randomized controlled trials that proved nature-based interventions can improve people’s depressive symptomology^7–9^. Nature-based recreation involves contact with nature, and it usually also includes physical activity and socialization—all of which may improve symptoms of depression, such as sad mood, sleep problems, difficulty concentrating, and feeling bad about yourself^5,8,10,11^. Our results, together with previous intervention studies, might be useful for practitioners as they suggest that people suffering from any symptom criterion from MDD will benefit from engaging in nature-based recreational activities by, for example, engaging on a twice-a-week basis. Thus, if confirmed in future RCTs, when treating MDD, therapists might suggest twice-a-week participation in forest-based activities, gardening, or nature-based adventure activities.

Due to the cross-sectional nature of our study, there are alternative ways to interpret the patterns of associations observed between nature-based recreation and depression^16,48,49^. Many studies have acknowledged the logical plausibility of a bidirectional relation between contact with nature and depression. This means that although activities in contact with nature can reduce depressive symptoms, severely depressed people may avoid participating in nature-based activities^50,51^. To our knowledge, however, empirical evidence largely supports one side of this causal reasoning; that is, evidence supporting contact with nature as a strategy to prevent or treat depression^7–9^. Nonetheless, the observed correlations might also reflect the possibility that severely depressed people might choose to avoid nature-based recreation. Another design limitation is the use of convenience samples. The use of a non-probabilistic sampling approach hinders the generalization of our results to wider populations but does not make it impossible^52^. The consistency of the negative correlation between nature-based recreation and depression shows that it is unlikely to be largely dependent on specific participants’ characteristics (e.g., gender, family income, and skin color), as can be observed in our sensitivity analyses. In the absence of evidence regarding the differential impact of nature-based recreation on depression based on people’s sociodemographic characteristics, we join previous calls for research exploring this issue^7–9^.

Given the prevalence of MDD, the variety of its symptomatology, and the potential value of nature-based interventions as a preventive strategy or treatment, we believe that further exploration of the effects of nature-based interventions on specific depressive symptoms is a fruitful line for future research. We have some suggestions related to this. First, existing experimental studies can reanalyze their data to assess the effect of nature-based interventions on specific depressive symptoms compared to usual care or alternative interventions. Second, future studies could build on our results to explore how different types of nature-based activities and different dosages affect depressive symptoms across unique countries. Third, new studies could run analyses both at the score and the symptom level, as we have done in the present study. Fourth, future systematic reviews can assess the effect of nature-based interventions on specific symptoms of MDD like anhedonia, feeling depressed or hopeless, sleep problems, and difficulty concentrating. These efforts would help to clarify the potential use of nature-based interventions to prevent or treat specific symptoms of depression as well as to prevent an MDD diagnosis or to help individuals achieve remission.

## Supplementary Information

Supplementary Information.

## Data Availability

The files with the data were submitted as supplementary files and the software used (RevMan) is free. Claudio D. Rosa can be contacted for questions related to the data analyzed in this study.
