# Supplementary material for: Associations between depression and nature-based recreation: A cross-sectional study of adults in the United States, Spain, and Brazil
Source: Sci Rep. 2025 Feb 10;15:4910. doi: 10.1038/s41598-025-89156-0 (PMC11811158; doi:10.1038/s41598-025-89156-0)
Supplement: Supplementary file 1 — Supplementary Information. [file 41598_2025_89156_MOESM1_ESM.docx]

**Associations between Depression and Nature-Based Recreation: A Cross-Sectional Study of Adults in the United States, Spain, and Brazil**

Claudio D. Rosa ¹, Lincol R. Larson ², Silvia Collado ³, *Sandra Geiger^4^, Christiana C. Profice^1^ and Marcos R. T. P. Menuchi^5^

¹ Department of Development and Environment, State University of Santa Cruz

² Department of Parks, Recreation and Tourism Management, North Carolina State University

³ Department of Psychology and Sociology, University of Saragossa

^4^ Department of Cognition, Emotion, and Methods in Psychology, University of Vienna

^5^ Department of Physical Education, State University of Santa Cruz

**Author Note**

Claudio D. Rosa
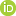
 <http://orcid.org/0000-0002-1939-2716>

Lincoln R. Larson
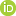
 <http://orcid.org/0000-0001-9591-1269>

Silvia Collado
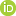
 <https://orcid.org/0000-0002-3905-0617>

Sandra Geiger
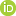
 <https://orcid.org/0000-0002-3262-5609>

Christiana C. Profice
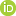
 <https://orcid.org/0000-0002-1972-9622>

Marcos R. T. P. Menuchi
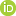
 <https://orcid.org/0000-0003-2833-7070>

**Supplementary File 1**

**Literature Review: Previous Observational Studies Assessing the Relation Between Contact with Nature and Depression**

To show the exact gap the present study fills in the existing knowledge and to be able to contextualize our results with previous studies, we conducted the broadest review (to date) of observational studies assessing the relation between contact with nature and depression. We considered relevant for this review (i.e., eligible studies) all observational studies that assessed the relation between visual or *in situ* contact with real nature (e.g., seeing nature through a window, visiting natural areas, or gardening) and depressive symptoms as assessed by a depression outcome measure (e.g., PHQ-9) or a physician diagnostic of depression. We did not consider relevant for this review studies that only assessed the existence of nature (e.g., distance from home to the nearest natural area) or that were not observational (i.e., assessed the impact of an intervention). Following the Cochrane Handbook for Systematic Reviews of Interventions, we did not exclude studies based on their date, publication status, language, or (selective) reporting of the results (Higgins et al., 2019). To illustrate, we excluded Das and Gailey (2022) because this study did not assess activities in contact with nature and excluded Olszewska-Guizzo et al. (2021) because this study describes an intervention provided to the research participants. Additionally, because it is not our intention to establish causality based on the included studies, we did not assess the studies' risk of bias formally (Antonakis et al., 2010; Higgins et al., 2019; Sterne et al., 2016, 2019; von Elm et al., 2007).

As a part of our search strategy, many recent systematic reviews assessing the relation between contact with nature and health-related outcomes were screened to find eligible studies (Astell-Burt et al., 2022; Barger et al., 2021; Beute et al., 2021; Britton et al., 2020; Browning et al., 2021; Coventry et al., 2021; Davis et al., 2021; Frost et al., 2022; Geneshka et al., 2021; Howarth et al., 2020; Jones et al., 2021; Kotera et al., 2021; Labib et al., 2020; Li et al., 2022; Marini et al., 2022; Nigg et al., 2021; Oswald et al., 2020; Rosa et al., 2021; Shin et al., 2020; Vibholm et al., 2020; Wilkie & Davinson, 2021; Williams et al., 2020; Yao et al., 2021; Yen et al., 2021; Yi et al., 2022; Zhang et al., 2020). Additionally, studies already known by our research team were considered since we have conducted systematic reviews on this field. Finally, the references from all eligible studies were checked to identify new relevant ones.

The first author of the present study conducted this process of selecting studies and extracted all relevant data from the studies he deemed eligible (Supplementary Table 1). As a result of this process, 33 observational studies were deemed eligible of which four have a longitudinal design and 29 a cross-sectional one. All studies were published in peer-reviewed journals from 2014 to 2023; most studies (73%) were published in the last five years 2019-2023. Studies involved a diversity of populations and countries but only two studies were conducted with children and adolescents (i.e., Gubbels et al., 2016; van Lier et al., 2017). Many forms of contact with nature were assessed including but not limited to visits to green spaces, gardening, nature walking, skiing, fishing, hiking, motorized nature-based activities, and park use. Also diverse were the measures used to assess depression. To cite a few, it encompassed the PHQ-2, PHQ-4, and PHQ-9, the depression subscale of the Depression, Anxiety, and Stress Scale (DASS)-21, and the Center for Epidemiologic Studies-Depression Scale-20.

Regarding the results from the included studies, virtually all studies found a negative correlation (not necessarily statistically significant) between contact with nature and depressive symptoms, denoting that a higher frequency or duration of contact with nature is associated with less frequent or intense depressive symptomatology (Supplementary Table 2). Only one study found a positive correlation between contact with nature and depression but the estimate was close to zero and imprecise (see Braçe et al., 2020 in Supplementary Table 2). Seven studies did not assess the relation between contact with nature and depression, though, they have the data to do so (Supplementary Table 2). Only six studies provided insights regarding a possible linear or “dose-response” relationship between contact with nature and depression (Supplementary Table 3). From these six studies, only two fully supported a “dose-response” relationship between contact with nature and depression.

After reviewing this body of evidence, we noted that (a) few studies analyzed the relation between depression and different kinds of activities in contact with nature. We also noted that (b) no previous study analyzed whether the relation between contact with nature and depression in its sample varies depending on the timeframe of the question (e.g., last 12 months vs. typical week). Moreover, (c) few studies provided information regarding the possibility of a “dose-response” relationship between contact with nature and depression. Finally, (d) no previous study analyzed the relation between contact with nature and the specific symptoms assessed by the depression outcome measures they used. Thus, the present study provides information regarding (a) to (d) research gaps.

**Supplementary Table 1**

*Characteristics of the 33 observational studies that collected data about contact with nature and depression and were included in our literature review*

| **Studies** | **Participants** | **n** | **Mean/median age or age range** | **%women** | **Country** | **Contact with nature** | **Depression measure** | **Design** | **Data collection**  **framework** |
| --- | --- | --- | --- | --- | --- | --- | --- | --- | --- |
| (Ricciardi et al., 2023) | South-Italian older adults | 454 | 73 | Not reported | Italy | Frequency of visits to greenspace | Geriatric Depression Scale −15 | Cross-sectional | Between January 2022 and April 2022 |
| (Zhang et al., 2023) | Adults (over age 18) living in Shanghai City | 386 | ≥ 18 | 66.6 | China | Gardening | Patient Health Questionnaire-9 | Cross-sectional | April-May 2022 |
| (Anderson et al., 2022) | Pregnant or less than 12 months postpartum women | 60 | 32.3 | 100.0 | United States | Spending time in nature | The Edinburgh Postnatal Depression Scale | Cross-sectional | Between April 2020 and April 2021 |
| (Bressane et al., 2022) | Adults living in Brazil | 1186 | ≥ 18 | Not reported | Brazil | Frequency of recreational or leisure activities in natural areas | Depression subscale of the Depression, Anxiety, and Stress Scale | Cross-sectional | June-July 2022 |
| (Quarta et al., 2022) Sample 1 | University students | 939 | ≥ 18 | 75.0 | Italy | Time spent in nature | Depression subscale of the Depression Anxiety Stress Scales-21 | Cross-sectional | From April to May 2021 |
| (Quarta et al., 2022) Sample 2 | University staff | 238 | ≥ 18 | 54.2 | Italy | Time spent in nature | Depression subscale of the Depression Anxiety Stress Scale-21 | Cross-sectional | From April to May 2021 |
| (Reid et al., 2022) | Denver residents | 801 | ≥ 18 | 58 | United States | Time spent in spaces with natural vegetation and at natural areas | Center for Epidemiologic Studies-Depression Scale-10 | Cross-sectional | Between November 16, 2019, and January 2, 2021 |
| (Samus et al., 2022) | Adults with access to a private garden | 261 | ≥ 18 | 84.3 | Germany and New Zealand | Time spent in the own garden and nature public spaces, and engagement in gardening activities | Center for Epidemiologic Studies-Depression Scale-20 | Cross-sectional | May 14 to May 24, 2020 |
| (Trevino et al., 2022) | University students who engaged in online learning during the COVID-19 outbreak | 353 | ≥ 18 | 77.4 | United States | Taking care of plants outside the home (Yes/No) | Depression subscale of the Depression Anxiety Stress Scale | Cross-sectional | Over two semesters in 2020 |
| (Basu et al., 2021) | Urban residents of India who have a home garden | 259 | Not reported | 53.7 | India | Time in the garden | Depression subscale of the Depression, Anxiety, and Stress Scale-21 | Cross-sectional | For two weeks at the end of May 2020 |
| (Bu et al., 2021) | Adults living in the United Kingdom | 55204 | ≥ 18 | 50 | United Kingdom | Gardening | Patient Health Questionnaire-9 | Longitudinal | Weekly during 11 weeks |
| (Corley et al., 2021) | Older adults who reported having access to the home garden | 171 | 84 | 47 | Scotland | Whether participants garden or relax and the frequency of garden usage | Hospital Anxiety and Depression Scale | Cross-sectional | The questionnaire was live between May 27 and June 8, 2020 |
| (Heo et al., 2021) | Adults living in South Korea | 322 | ≥ 19 | 76.2 | South Korea | Green space visits | Patient Health Questionnaire-9 | Cross-sectional | September–December 2020 |
| **Studies** | **Participants** | **n** | **Mean/median age or age range** | **%women** | **Country** | **Contact with nature** | **Depression measure** | **Design** | **Data collection**  **framework** |
| (Hubbard et al., 2021) | People aged 16 or older, able to speak English, and currently living in Scotland | 502 | 53 | 11.6 | Scotland | Frequency and duration of visits to green space | Patient Health Questionnaire -4 | Cross-sectional | One week at the beginning of July 2020 |
| (Li et al., 2021) | Chinese male prisoners | 269 | 34.5 | 0.0 | China | Viewing nature through a window | Patient Health Questionnaire-9 | Cross-sectional | March 3, 2021 |
| (Lõhmus et al., 2021) | Adults living in Stockholm County | 2060 | ≥20 | 55.3 | Sweden | Visit nature areas | Depression subscale of the Hopkins Symptom Checklist | Cross-sectional | Between 5 June and 1 August 2020 |
| (Marques et al., 2021) | Adults living in the city of Rio de Janeiro | 173 | ≥ 18 | 77 | Brazil | Visiting green areas and taking care of plants | Depression subscale of the Depression, Anxiety, and Stress Scale-21 | Cross-sectional | November 6th 2020 to January 24th 2021 |
| (Pouso et al., 2021) | Adults from countries with at least 100 participants | 5218 | ≥ 18 | 65.2 | Nine countries^a^ | Time spent per day in natural spaces | Patient Health Questionnaire-2 | Cross-sectional | Between 17th April and 8th May 2020 |
| (Braçe et al., 2020) | Adults with at least one year of residence in their current home | 446 | 43.0 | 50.1 | Spain | Frequency of green spaces visits | Hospital Anxiety and Depression Scale (HADS) | Cross-sectional | Two periods: from February to May and September to November 2018 |
| (Tomasi et al., 2020) | Adults diagnosed with depression or anxiety disorders | 59 | 40.3 | 57.6 | Spain and Italy | Aggregated score from five nature-based and one outdoor activity | Beck Depression Inventory-II | Cross-sectional | Not reported |
| (Marselle et al., 2019) | Adults who attended at least one nature group walk | 1516 | ≥ 18 | 66.2 | United Kingdom | Frequency nature group walkers were compared to the non-frequent ones | The 10-item Major Depressive Inventory | Longitudinal | T0: First group walk;  T1: Baseline mental health;  T2: Three months after T1. |
| (Svensson et al., 2019) | Skiers (participants in a race) and matched non-skiers from the general population | 395369 | 36 | 38 | Sweden | Participating in a skiing event and time to complete the race | Diagnostic of depressive disorders from the Swedish National Patient Registry | Longitudinal | Up to 21 years follow-up. Median follow-up of 10 years. |
| (Wolsko et al., 2019) | Adults enrolled in the Amazon MTurk system | 231 | 37.8 | 56.9 | United States | Seven sets of nature-based activities (e.g., hiking, fishing, and non-motorized boating) | Zung Self-Rating Depression Scale | Cross-sectional | Not reported |
| (Wood et al., 2019) | Adults | 310 | 33.1 | 54.3 | United Kingdom | Frequency of past and actual exposure to nature-rich environments | Depression subscale of the Depression, Anxiety, and Stress Scale-21 | Cross-sectional | Unclear |
| (Cox et al., 2018) | Adults across the United Kingdom | 3000 | 18 to 70 | 57.4 | United Kingdom | Frequency and duration of garden and public green spaces visits | Depression subscale of the Depression, Anxiety, and Stress Scale-21 | Cross-sectional | Over two weeks in May 2016 |
| (Cox et al., 2017) | Adults from the Cranfield triangle | 1023 | ≥ 18 | 53 | United Kingdom | Frequency and duration of time spent in own garden | Depression subscale of the Depression, Anxiety, and Stress Scale-21 | Cross-sectional | Over two weeks in May 2014 |
| **Studies** | **Participants** | **n** | **Mean/median age or age range** | **%women** | **Country** | **Contact with nature** | **Depression measure** | **Design** | **Data collection**  **framework** |
| (van Lier et al., 2017) | Secondary school students | 8500 | 12 to 18 years old | 53.7 | New Zealand | Home gardening | Reynolds Adolescents Depression Scale-Short Form | Cross-sectional | National Youth Health Survey conducted in 2012 |
| (Gubbels et al., 2016) Study 1 | Adolescents from severely deprived districts | 401 | 13.2 | 46.3 | Netherlands | Physical activity in the green space living environment, in the past year | Center for Epidemiologic Studies-Depression Scale-20 | Longitudinal | T0: Between May 2010 and May 2011;  T1: Between May and July 2012. |
| (Gubbels et al., 2016) Study 2 | Adults from severely deprived districts | 454 | 46.3 | 55.7 | Netherlands | Physical activity in the green space living environment, in the past year | Center for Epidemiologic Studies-Depression Scale-20 | Longitudinal | T0: Between May 2010 and May 2011  T1: Between May and July 2012 |
| (Shanahan et al., 2016) | Residents of Brisbane City, Australia | 1538 | 18 to 70 | Not reported | Australia | Frequency and duration of visits to green spaces | Depression subscale of Depression, Anxiety, and Stress Scale-21 | Cross-sectional | November 2012 |
| (Snell et al., 2016) | Adults citizens of or currently living in Australia | 300 | 33.8 | 54.7 | Australia | Frequency of contact with nature in childhood and adulthood | Depression subscale of Depression, Anxiety, and Stress Scale-21 | Cross-sectional | Not reported |
| (Torres et al., 2016) | Non-depressed African Americans | 2903 | 42 | 63.5 | United States | Frequency of gardening/yard work | Center for Epidemiologic Studies-Depression Scale-12 items | Cross-sectional | Cross-sectional interviews were conducted in 2001-2003 |
| (Reklaitiene et al., 2014) | Urban population | 5009 | 46-72 | 54.7 | Lithuania | Park use | Center for Epidemiologic Studies Depression Scale-10 items | Cross-sectional | Between 2006 and 2008 |

*Note***.** ^a^Spain, United Kingdom, Germany, France, United States, Portugal, Italy, New Zealand and Mexico.

**Supplementary Table 2**

*The direction of the relation between contact with nature and depression observed by the 33 observational studies included in our literature review*

| First author (year) | Negative association between contact with nature and depression | Positive association between contact with nature and depression | The association between contact with nature and depression was not assessed |
| --- | --- | --- | --- |
| (Ricciardi et al., 2023) | Frequency and duration | - | - |
| (Zhang et al., 2023) | Frequency | - | - |
| (Anderson et al., 2022) | Impossible to discern the frequency and duration | - | - |
| (Bressane et al., 2022) | Frequency | - | - |
| (Quarta et al., 2022) Sample 1 | Frequency | - | - |
| (Quarta et al., 2022) Sample 2 | Frequency | - | - |
| (Reid et al., 2022) | Impossible to discern the frequency and duration | - | - |
| (Samus et al., 2022) | Duration | - | - |
| (Trevino et al., 2022) | - | - | Did not assess |
| (Basu et al., 2021) | Duration | - | - |
| (Bu et al., 2021) | Duration | - | - |
| (Corley et al., 2021) | Impossible to discern the frequency and duration | - | - |
| (Heo et al., 2021) | Frequency | - | - |
| (Hubbard et al., 2021) | - | - | Did not assess |
| (Li et al., 2021) | Frequency and duration | - | - |
| (Lõhmus et al., 2021) | - | - | Did not assess |
| (Marques et al., 2021) | - | - | Did not assess |
| (Pouso et al., 2021) | - | - | Did not assess |
| (Braçe et al., 2020) | - | Frequency | - |
| (Tomasi et al., 2020) | Frequency | - | - |
| (Marselle et al., 2019) | Frequency | - | - |
| (Svensson et al., 2019) | Frequency | - | - |
| (Wolsko et al., 2019) | Frequency | - | - |
| (Wood et al., 2019) | Frequency | - | - |
| (Cox et al., 2018) | Frequency and duration | - | - |
| (Cox et al., 2017) | Frequency and duration | - | - |
| (van Lier et al., 2017) | Impossible to discern the frequency and duration | - | - |
| (Gubbels et al., 2016) Study 1 | - | - | Did not assess |
| (Gubbels et al., 2016) Study 2 | - | - | Did not assess |
| (Shanahan et al., 2016) | Frequency and duration | - | - |
| (Snell et al., 2016) | Frequency | - | - |
| (Torres et al., 2016) | Frequency | - | - |
| (Reklaitiene et al., 2014) | - | - | Did not assess |

**Supplementary Table 3**

*Main findings of previous observational studies that assessed “dose-response” relationships between contact with nature and depression*

| Studies | Main findings |
| --- | --- |
| (Reid et al., 2022) | All groups who had a greater agreement with the sentence “I spend a lot of time in spaces with natural vegetation” held a lower depression score than groups with a lower agreement with this sentence, suggesting a “dose-response” relationship. |
| (Bressane et al., 2022) | The study found support for a “dose-response” relationship because all groups with a higher frequency of engagement in recreational or leisure activities in natural areas held a lower prevalence of depression than groups with a lower frequency of engagement in these activities. |
| (Heo et al., 2021) | No support for a “dose-response” relationship was found. Participants who visited green spaces 1 to 2 times a week had lower odds of major depression than participants who visited these spaces 0-2 times per month. Nonetheless, the odds of depression were higher for individuals who visited green spaces more frequently than 1 to 2 times a week. |
| (Marselle et al., 2019) | No support for a “dose-response” relationship was found. The association between attending at least once a nature group walking with depression was similar to the association between attending this group weekly and depression. |
| (Cox et al., 2017) | This study did not fully support a “dose-response” relationship. Individuals who spent more than 10 minutes in their garden two or more times in the last week held a lower prevalence of depression than individuals who did it less frequently. Nonetheless, individuals who spent more than 10 minutes in their garden once last week had a higher prevalence of depression than the individuals who did it less frequently. The results were even less straightforward for the duration of time spent on own garden. |
| (Shanahan et al., 2016) | This study did not fully support a “dose-response” relationship. There was a tendency to observe a lower prevalence of depression among groups who spent more time in green space visits during the survey week compared to individuals who spent less time in these visits, but this tendency was not linear. |

**Supplementary Table 4**

*Percentage of participants reporting their frequency of nature-based recreation in the past 12 months*

|  | United States of America | | | | Spain | | | | Brazil | | | | Overall | | | |
| --- | --- | --- | --- | --- | --- | --- | --- | --- | --- | --- | --- | --- | --- | --- | --- | --- |
|  | **Any activity** | **Forest-based** | **Gardening** | **Adventure** | **Any activity** | **Forest-based** | **Gardening** | **Adventure** | **Any activity** | **Forest-based** | **Gardening** | **Adventure** | **Any activity** | **Forest-based** | **Gardening** | **Adventure** |
| Never | 5.3 | 7.3 | 36.9 | 17.4 | 5.5 | 8.0 | 32.8 | 15.8 | 8.7 | 24.1 | 41.5 | 39.1 | 6.4 | 12.6 | 37.1 | 23.4 |
| Rarely | 19.0 | 24.1 | 31.2 | 38.7 | 27.2 | 31.9 | 39.7 | 38.4 | 43.1 | 48.0 | 29.8 | 41.3 | 28.6 | 33.6 | 33.3 | 39.4 |
| Sometimes | 28.2 | 30.1 | 14.7 | 24.0 | 29.0 | 30.5 | 13.3 | 26.5 | 22.3 | 16.7 | 11.0 | 10.6 | 26.7 | 26.2 | 13.2 | 20.7 |
| Often | 26.7 | 23.8 | 9.9 | 13.9 | 25.8 | 20.4 | 9.2 | 14.0 | 16.3 | 7.4 | 9.0 | 4.9 | 23.3 | 17.9 | 9.4 | 11.2 |
| Very often | 20.8 | 14.7 | 7.3 | 6.1 | 12.6 | 9.2 | 5.0 | 5.3 | 9.6 | 3.8 | 8.7 | 4.0 | 15.0 | 9.8 | 7.1 | 5.2 |
| At least monthly | 75.7 | 68.6 | 31.9 | 44.0 | 67.4 | 60.1 | 27.5 | 45.8 | 48.2 | 27.9 | 28.7 | 19.6 | 65.0 | 53.9 | 29.7 | 37.2 |

*Note.* Never*:* I never participated; Rarely: I rarely participated (a few times a year); Sometimes: I sometimes participated (about once a month); Often: I often participated (several times each month); Very often: I very often participated (pretty much every week).

**Supplementary Table 5**

*Percentage of participants reporting their frequency of nature-based recreation in a typical week*

|  | United States of America | | | | Spain | | | | Brazil | | | | Overall | | | |
| --- | --- | --- | --- | --- | --- | --- | --- | --- | --- | --- | --- | --- | --- | --- | --- | --- |
|  | **Any activity** | **Forest-based** | **Gardening** | **Adventure** | **Any activity** | **Forest-based** | **Gardening** | **Adventure** | **Any activity** | **Forest-based** | **Gardening** | **Adventure** | **Any activity** | **Forest-based** | **Gardening** | **Adventure** |
| None | 19.0 | 25.5 | 62.0 | 53.1 | 20.5 | 31.7 | 62.9 | 45.9 | 38.6 | 58.2 | 62.3 | 68.2 | 25.3 | 37.1 | 62.4 | 55.5 |
| 1 day | 32.2 | 42.3 | 23.0 | 32.1 | 32.9 | 35.8 | 23.0 | 32.3 | 32.8 | 30.0 | 20.9 | 21.3 | 32.6 | 36.7 | 22.4 | 28.9 |
| 2 days | 18.5 | 14.0 | 5.8 | 7.9 | 21.5 | 15.8 | 6.5 | 12.4 | 12.9 | 5.4 | 6.1 | 4.3 | 17.7 | 12 | 6.1 | 8.1 |
| 3 days | 13.2 | 9.6 | 4.8 | 3.6 | 11.9 | 8.7 | 3.7 | 3.9 | 6.7 | 2.2 | 2.7 | 2.7 | 10.9 | 7.1 | 3.8 | 3.4 |
| 4 days | 8.6 | 4.1 | 2.1 | 1.3 | 6.2 | 3.9 | 1.8 | 3.2 | 4.2 | 1.6 | 2.9 | 1.3 | 6.6 | 3.3 | 2.3 | 1.9 |
| 5 days | 5.3 | 1.7 | 0.7 | 1.2 | 4.8 | 2.3 | 1.4 | 1.2 | 0.9 | 0.4 | 1.3 | 0.9 | 3.8 | 1.5 | 1.1 | 1.1 |
| 6 days | 0.8 | 1.5 | 0.3 | 0.2 | 0.7 | 0.5 | 0.2 | 0.7 | 1.3 | 0.4 | 0.9 | 0.7 | 0.9 | 0.9 | 0.5 | 0.5 |
| Every day | 2.5 | 1.3 | 1.3 | 0.7 | 1.6 | 1.4 | 0.5 | 0.5 | 2.5 | 1.8 | 2.9 | 0.7 | 2.2 | 1.5 | 1.5 | 0.6 |
| At least one day | 81.0 | 74.5 | 38.0 | 46.9 | 79.5 | 68.3 | 37.1 | 54.1 | 61.4 | 41.8 | 37.7 | 31.8 | 74.7 | 62.9 | 37.6 | 44.5 |

**Supplementary Table 6**

*PHQ-9 mean, Standard Deviation, Minimum, Maximum, Skewness, and Kurtosis from the North American, Spanish, and Brazilian Samples*

|  | Mean | Standard deviation | Minimum | Maximum | Skewness | Kurtosis |
| --- | --- | --- | --- | --- | --- | --- |
| **United States of America** | 7.83 | 5.69 | 0 | 26 | 0.87 | 0.23 |
| **Spanish** | 7.12 | 4.78 | 0 | 27 | 1.18 | 2.01 |
| **Brazil** | 11.09 | 6.63 | 0 | 27 | 0.44 | -0.62 |

**Supplementary Table 7**

*Percentage of participants who reported being bothered by depressive symptoms over the last two weeks*

|  | United States of America | | | | Spain | | | | Brazil | | | | Overall | | | |
| --- | --- | --- | --- | --- | --- | --- | --- | --- | --- | --- | --- | --- | --- | --- | --- | --- |
|  | **Not at all** | **Several days** | **More than half the days** | **Nearly every day** | **Not at all** | **Several days** | **More than half the days** | **Nearly every day** | **Not at all** | **Several days** | **More than half the days** | **Nearly every day** | **Not at all** | **Several days** | **More than half the days** | **Nearly every day** |
| Little interest or pleasure in doing things | 32.6 | 46.4 | 14.2 | 6.8 | 29.2 | 53.9 | 13.9 | 3.0 | 13.6 | 43.1 | 21.2 | 22.1 | 25.9 | 47.6 | 16.2 | 10.3 |
| Feeling down depressed or hopeless | 41.4 | 39.4 | 11.9 | 7.3 | 37.4 | 49.8 | 8.2 | 4.6 | 24.1 | 41.1 | 17.9 | 17.0 | 35.1 | 43 | 12.6 | 9.4 |
| Trouble falling or staying asleep or sleeping too much | 29.4 | 36.1 | 20.3 | 14.2 | 35.8 | 37.7 | 15.8 | 10.7 | 22.1 | 35.5 | 19.0 | 23.4 | 29.1 | 36.4 | 18.6 | 16 |
| Feeling tired or having little energy | 13.0 | 48.7 | 22.6 | 15.7 | 16.0 | 54.3 | 19.4 | 10.3 | 8.3 | 34.6 | 25.9 | 31.3 | 12.5 | 46.1 | 22.7 | 18.8 |
| Poor appetite or overeating | 41.6 | 33.2 | 14.4 | 10.9 | 35.6 | 37.4 | 17.1 | 9.8 | 32.4 | 28.1 | 19.4 | 20.1 | 37.1 | 32.9 | 16.7 | 13.3 |
| Feeling bad about yourself or that you are a failure | 43.1 | 35.8 | 12.7 | 8.4 | 50.2 | 37.2 | 8.7 | 3.9 | 37.3 | 28.3 | 15.8 | 18.5 | 43.4 | 34 | 12.5 | 10.1 |
| Trouble concentrating | 38.9 | 35.6 | 14.4 | 11.1 | 40.0 | 43.8 | 12.3 | 3.9 | 24.6 | 30.1 | 21.4 | 23.9 | 34.9 | 36.4 | 15.9 | 12.8 |
| Agitation or retardation | 70.6 | 19.1 | 7.1 | 3.1 | 61.2 | 29.5 | 7.3 | 2.1 | 50.0 | 26.8 | 13.8 | 9.4 | 61.7 | 24.5 | 9.2 | 4.7 |
| Suicidal ideation | 83.6 | 11.4 | 4.0 | 1.0 | 87.6 | 8.7 | 2.1 | 1.6 | 79.0 | 10.9 | 5.4 | 4.7 | 83.4 | 10.5 | 3.8 | 2.3 |

**Supplementary** **Table 8**

*PHQ-9 scores according to the frequency of participation in nature-based recreation during the last 12 months*

|  | United States of America | | | | Spain | | | | Brazil | | | | Overall | | | |
| --- | --- | --- | --- | --- | --- | --- | --- | --- | --- | --- | --- | --- | --- | --- | --- | --- |
|  | **Any activity** | **Forest-based** | **Gardening** | **Adventure** | **Any activity** | **Forest-based** | **Gardening** | **Adventure** | **Any activity** | **Forest-based** | **Gardening** | **Adventure** | **Any activity** | **Forest-based** | **Gardening** | **Adventure** |
| Never | 8.88 | 9.59 | 8.77 | 9.28 | 6.75 | 9.09 | 7.34 | 8.07 | 15.03 | 13.12 | 12.45 | 12.72 | 10.86 | 11.53 | 9.64 | 10.76 |
| Rarely | 10.78 | 9.83 | 7.37 | 8.83 | 7.63 | 7.26 | 7.47 | 7.12 | 12.48 | 11.56 | 11.48 | 10.91 | 10.67 | 9.86 | 8.52 | 8.99 |
| Sometimes | 8.31 | 7.75 | 7.66 | 6.69 | 7.87 | 7.39 | 6.10 | 6.92 | 10.94 | 9.61 | 9.16 | 10.19 | 8.83 | 7.99 | 7.58 | 7.31 |
| Often | 6.51 | 6.97 | 7.03 | 6.64 | 6.36 | 6.20 | 7.53 | 7.25 | 8.67 | 7.12 | 8.95 | 6.95 | 6.92 | 6.73 | 7.72 | 6.90 |
| Very often | 5.88 | 5.29 | 6.55 | 4.65 | 6.02 | 6.30 | 5.32 | 5.13 | 5.74 | 6.47 | 7.95 | 5.06 | 5.89 | 5.71 | 6.81 | 4.88 |

*Note.* Never: I never participated; Rarely: I rarely participated (a few times a year); Sometimes: I sometimes participated (about once a month); Often: I often participated (several times each month); Very often: I very often participated (pretty much every week).

**Supplementary Table 9**

*PHQ-9 scores according to the frequency of participation in nature-based recreation during a typical week*

|  | United States of America | | | | Spain | | | | Brazil | | | | Overall | | | |
| --- | --- | --- | --- | --- | --- | --- | --- | --- | --- | --- | --- | --- | --- | --- | --- | --- |
|  | **Any activity** | **Forest-based** | **Gardening** | **Adventure** | **Any activity** | **Forest-based** | **Gardening** | **Adventure** | **Any activity** | **Forest-based** | **Gardening** | **Adventure** | **Any activity** | **Forest-based** | **Gardening** | **Adventure** |
| None | 9.88 | 9.68 | 8.02 | 8.85 | 7.38 | 7.22 | 7.27 | 7.37 | 13.85 | 12.22 | 12.07 | 12.06 | 11.10 | 10.26 | 9.02 | 9.68 |
| 1 day | 9.04 | 8.09 | 7.97 | 6.71 | 7.49 | 7.63 | 7.41 | 7.36 | 10.63 | 10.16 | 10.39 | 10.27 | 9.06 | 8.47 | 8.48 | 7.71 |
| 2 days | 6.38 | 5.71 | 7.11 | 7.34 | 7.23 | 6.72 | 6.57 | 6.57 | 8.52 | 8.46 | 9.56 | 7.21 | 7.15 | 6.47 | 7.68 | 6.97 |
| 3 days | 5.67 | 5.74 | 5.90 | 5.82 | 6.22 | 6.89 | 6.81 | 4.24 | 9.17 | 7.60 | 8.92 | 8.00 | 6.50 | 6.33 | 6.79 | 5.80 |
| 4 days | 6.56 | 7.16 | 7.83 | 5.50 | 5.63 | 4.71 | 3.25 | 6.21 | 6.58 | 10.00 | 7.92 | 5.17 | 6.31 | 6.71 | 6.76 | 5.79 |
| 5 days | 5.53 | 5.10 | 4.75 | 6.57 | 6.67 | 6.40 | 4.83 | 13.20 | 6.75 | 4.50 | 10.67 | 5.00 | 6.04 | 5.64 | 7.00 | 8.25 |
| 6 days | 10.80 | 8.00 | 8.50 | 0.00 | 9.33 | 12.50 | 1.00 | 4.67 | 4.33 | 4.00 | 4.00 | 2.67 | 7.71 | 8.08 | 4.86 | 3.14 |
| Every day | 6.93 | 6.50 | 7.50 | 5.25 | 7.43 | 5.33 | 11.00 | 4.50 | 5.73 | 7.50 | 6.38 | 6.33 | 6.64 | 6.55 | 7.17 | 5.44 |

**Supplementary Table 10**

*Fixed-effects Meta-analyses Estimating the Prevalence Ratio of Holding a PHQ-9 Score ≥ 10 According to Montly Participation in Any Nature-Based Recreation Activity. Estimates are Based on Data from All Participants*

| Men | | | Women | | |
| --- | --- | --- | --- | --- | --- |
| Prevalence Ratio | Lower boundary of the 95%CI | Higher boundary of the 95%CI | Prevalence Ratio | Lower boundary of the 95%CI | Higher boundary of the 95%CI |
| 2.47 | 1.90 | 3.21 | 1.60 | 1.37 | 1.86 |
| Up to average family income | | | More than average family income | | |
| Prevalence Ratio | Lower boundary of the 95%CI | Higher boundary of the 95%CI | Prevalence Ratio | Lower boundary of the 95%CI | Higher boundary of the 95%CI |
| 1.99 | 1.69 | 2.33 | 1.42 | 1.11 | 1.83 |
| Whiter skin color (2 or less in the NIS Skin Color Scale) | | | Darker skin color (3 or more in the NIS Skin Color Scale) | | |
| Prevalence Ratio | Lower boundary of the 95%CI | Higher boundary of the 95%CI | Prevalence Ratio | Lower boundary of the 95%CI | Higher boundary of the 95%CI |
| 1.70 | 1.42 | 2.02 | 1.92 | 1.57 | 2.36 |

*Note.* A Prevalence Ratio greater than 1 indicates that participants who were not engaged at least monthly during the last 12 months in any nature-based recreation activities were more likely to hold a PHQ-9 score *≥ 10* than the participants who did engage in those activities more frequently.

**Supplementary Figure 1**

*Fixed-Effects Meta-Analyses of the Mean Difference in PHQ-9 scores (95% CI) According to Monthly Participation in Nature-Based Recreation. Estimates are Based on Combined Data from the North Americans, Spanish, and Brazilian Participants. (a) Refers to any Nature-Based Recreational Activity. (b) Refers to Forest-Based Activities. (c) Refers to Gardening. (d) Refers to Nature-Based Adventure.*


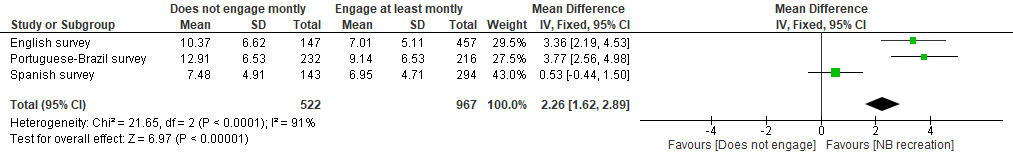


(a)


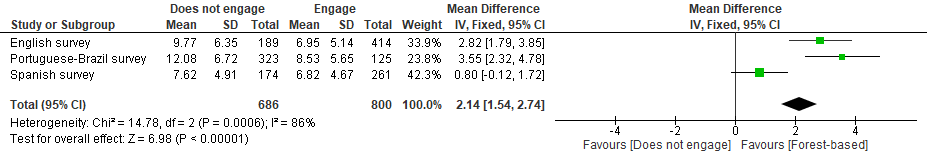


(b)


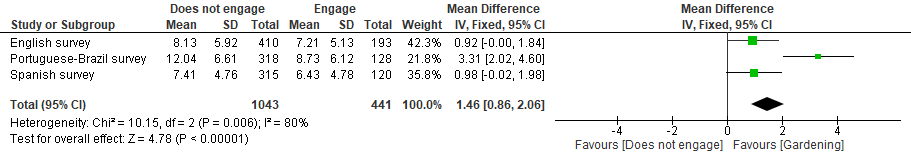


(c)


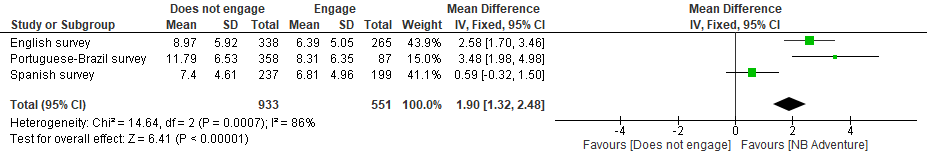


(d)

**Supplementary Figure 2**

*Fixed-Effects Meta-Analyses of the Mean Difference in PHQ-9 scores (95% CI) According to Weekly Participation in Nature-Based Recreation. Estimates are Based on Combined Data from the North Americans, Spanish, and Brazilian Participants. (a) Refers to any Nature-Based Recreational Activity. (b) Refers to Forest-Based Activities. (c) Refers to Gardening. (d) Refers to Nature-Based Adventure.*


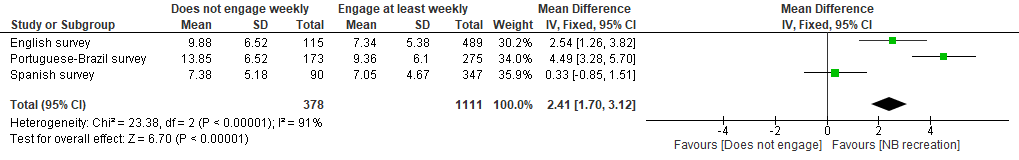


(a)


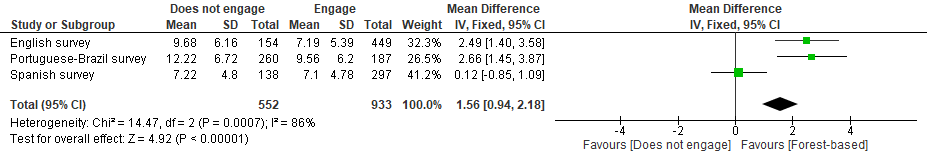


(b)


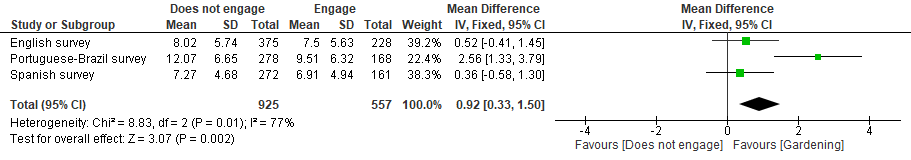


(c)


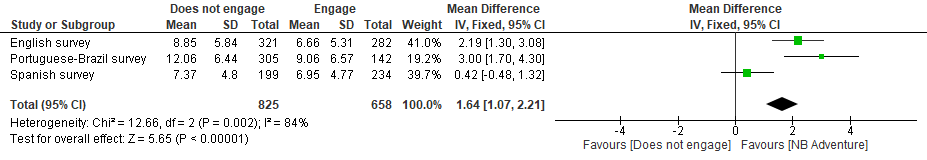


(d)

**Supplementary Figure 3**

*Fixed-Effects Meta-Analyses Estimating the Prevalence Ratio of Holding a PHQ-9 score ≥ 10 According to Monthly Participation in Nature-Based (NB) Recreation. Estimates are Based on Data From the the North Americans, Spanish, and Brazilian Participants. Events Refer to the Number of Participants with a PHQ-9 score ≥ 10. (a) Refers to any NB Recreational Activity. (b) Refers to Forest-Based Activities. (c) Refers to Gardening. (d) Refers to NB Adventure*


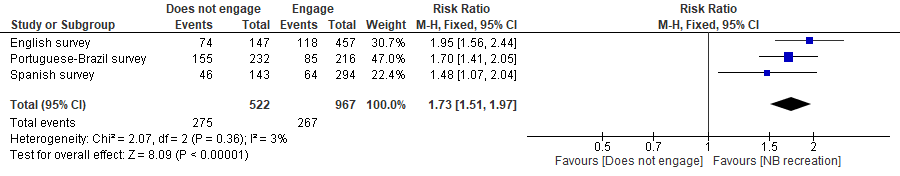


(a)

**
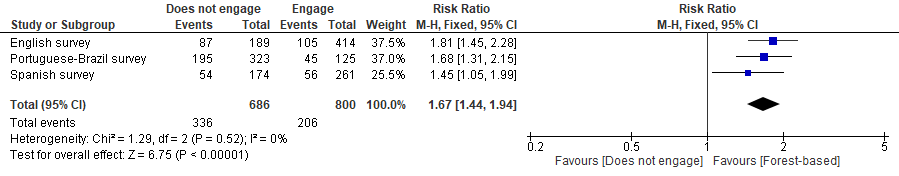
**

(b)

**
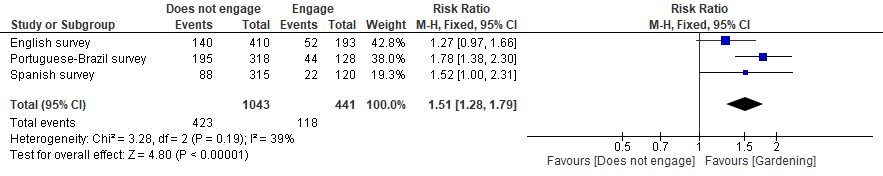
**

(c)

**
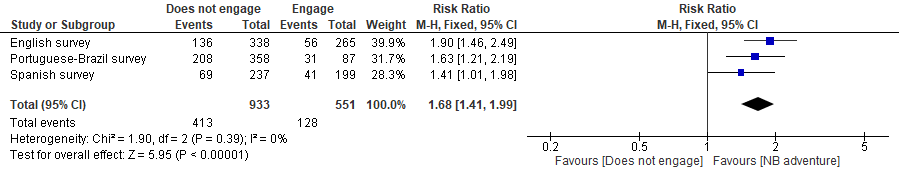
**

(d)

**Supplementary Figure 4**

*Fixed-Effects Meta-Analyses Estimating the Prevalence Ratio of Holding a PHQ-9 score ≥ 10 According to Weekly Participation in Nature-Based Recreation. Estimates are Based on Data From the the North Americans, Spanish, and Brazilian Participants. Events Refer to the Number of Participants with a PHQ-9 score ≥ 10. (a) Refers to any Nature-Based Recreational Activity. (b) Refers to Forest-Based Activities. (c) Refers to Gardening. (d) Refers to Nature-Based Adventure*

**
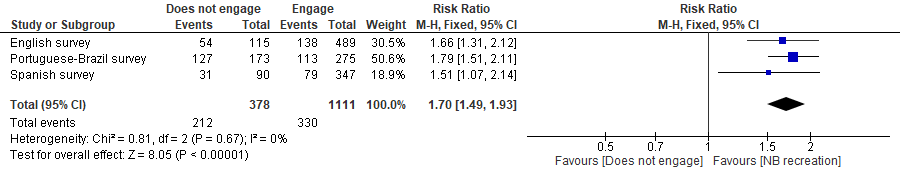
**

(a)

**
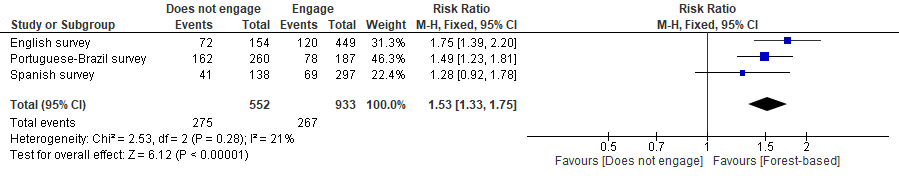
**

(b)

**
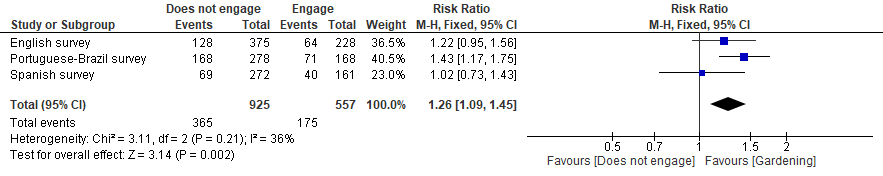
**

(c)

**
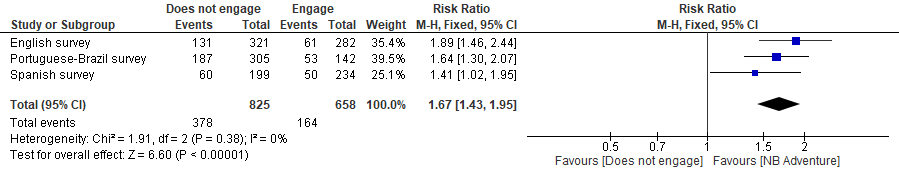
**

(d)

**QUESTIONNAIRE SURVEY IN ENGLISH, SPANISH, AND PORTUGUESE**

**ENGLISH VERSION**

**Nature and Mental Health Survey**
 Dear Participant,

 Welcome to this study.

 **Free and Informed Consent Form**
 We hereby invite you to participate, as a volunteer, in a survey that aims to understand the frequency of participation in activities in contact with nature and the occurrence of depressive symptoms in people. This research is entitled: Activities in contact with nature and symptoms of depression in adults. Your participation in this study will enhance our understanding of possible connections between time in nature and the feelings/emotions that people can experience.

 To explore these connections, you will answer a questionnaire on the following pages with questions regarding activities in contact with nature and some symptoms of depression. **Completing the questionnaire should only take 5-10 minutes.**

 All information collected will be used exclusively for research purposes and anonymity will be guaranteed. To prevent your identity from being revealed, only the responsible researchers will have access to information that allows your identification, such as your email address. In addition, you may experience some psychological disturbance, such as tiredness, when answering the questionnaire. You may stop completing the questionnaire at any time and for any reason, without any penalty, even after agreeing to participate. Your participation will not incur any financial cost to participate. If you have any loss that justifies damages and reparations, you will have the right to claim compensation. If there is any cost, you will be reimbursed.

 You can contact the leading researchers of this study if you need any clarification or assistance, free of charge and for as long as necessary. After participating in the survey, you will receive an individual report with your information and the results. If you agree to participate in this study, the data recorded and some information such as sociodemographic data (gender, age, and education) will be used solely for teaching and research. Additionally, a copy of this form, signed by the leading researcher, can be downloaded at the following link https://osf.io/tk8wx.
   **Lead researcher:
 Brazil** Claudio Damião Rosa (Ph.D. student)
 cdrosa@uesc.br
 55 73 991123063
 State University of Santa Cruz **Lead researcher:
 United States** Dr. Lincoln Larson (Associate Professor)
 LRLarson@ncsu.edu
 1-919-515-8947
 North Carolina State University

**Please answer the prompt below if you agree to participate in the study.**

- I understand what the research is about and I agree to participate.

Country In which country do you currently live?

- Brazil (1)
- Spain (2)
- United States (3)
- Other (please specify): (4) __________________________________________________

Text In this section, you will find some questions related to how you have been feeling in the last 2 weeks.

WHO-5 Please indicate for each of the 5 statements which is closest to how you have been feeling over the past 2 weeks.

 Over the past 2 weeks...

|  | At no time (0) | Some of the time (1) | Less than half of the time (2) | More than half of the time (3) | Most of the time (4) | All of the time (5) |
| --- | --- | --- | --- | --- | --- | --- |
| ... I have felt cheerful and in good spirits (Cheerful) |  |  |  |  |  |  |
| ... I have felt calm and relaxed (Calm) |  |  |  |  |  |  |
| ... I have felt active and vigorous (Active) |  |  |  |  |  |  |
| ... I woke up feeling fresh and rested (Rested) |  |  |  |  |  |  |
| ... my daily life has been filled with things that interest me (Interested) |  |  |  |  |  |  |

|  |  |
| --- | --- |

Symptoms1 Over the last 2 weeks, how often have you been bothered by any of the following problems?

|  | Not at all (0) | Several days (1) | More than half the days (2) | Nearly every day (3) |
| --- | --- | --- | --- | --- |
| Little interest or pleasure in doing things (Pleasure) |  |  |  |  |
| Feeling down, depressed, or hopeless (Depressed) |  |  |  |  |
| Trouble falling or staying asleep or sleeping too much (Sleep) |  |  |  |  |
| Feeling tired or having little energy (Tired) |  |  |  |  |
| Poor appetite or overeating (Appetite) |  |  |  |  |

|  |  |
| --- | --- |

Symptoms2 Over the last 2 weeks, how often have you been bothered by any of the following problems?

|  | Not at all (0) | Several days (1) | More than half the days (2) | Nearly every day (3) |
| --- | --- | --- | --- | --- |
| Feeling bad about yourself—or that you are a failure or have let yourself or your family down (Failure) |  |  |  |  |
| Trouble concentrating on things, such as reading the newspaper or watching television (Concentration) |  |  |  |  |
| Moving or speaking so slowly that other people could have noticed. Or the opposite—being so fidgety or restless that you have been moving around a lot more than usual (Slow speech) |  |  |  |  |
| Thoughts that you would be better off dead or of hurting yourself (Thoughs) |  |  |  |  |

|  |  |
| --- | --- |

Difficulty If you checked off any problems, how difficult have these problems made it for you to do your work, take care of things at home, or get along with other people?

- I did not check off any problem (99)
- Not difficult at all (1)
- Somewhat difficult (2)
- Very difficult (3)
- Extremely difficult (99)

| Page Break |  |
| --- | --- |

|  |  |
| --- | --- |

Treatment Which of the following treatments, if any, are you currently using to improve your mental health? (Check ALL that apply.)

- No treatment (0)
- Psychotherapy and/or counseling (1)
- Medicine(s) prescribed by a doctor (2)
- Other (please specify): (3) __________________________________________________

Text Here, you will find a few questions about your participation in nature-based recreation and leisure activities. We are interested in different types of nature-based activities and how often you do them, so please read each question carefully.

 While replying to these questions consider that nature-based recreation and leisure activities are activities **in contact with nature** that you choose to do during your free time. Examples of these activities are visiting parks, hiking on trails/walking, reading in nature, swimming in a lake or ocean, and gardening.

|  |  |
| --- | --- |

Yearly contact First, tell us about your *general* participation in nature-based recreation and leisure activities.
 In the past 12 months, which of the following best describes your participation in **ANY type of nature-based recreation and leisure activities**?

- I **never** participated (0)
- I **rarely** participated (a few times a year) (1)
- I **sometimes** participated (about once a month) (2)
- I **often** participated (several times each month) (3)
- I **very often** participated (pretty much every week) (4)

| Page Break |  |
| --- | --- |

|  |  |
| --- | --- |

Yearly activities Next, tell us about your participation in *specific types* of nature-based recreation and leisure activities. In the past 12 months, how often have you engaged in the following activities?

|  | I never participated (0) | I rarely participated (a few times a year) (1) | I sometimes participated (about once a month) (2) | I often participated (several times each month) (3) | I very often participated (pretty much every week) (4) |
| --- | --- | --- | --- | --- | --- |
| Activities in forested areas (such as parks or any other areas with many trees) (Yearly forest) |  |  |  |  |  |
| Activities related to gardening (such as planting and caring for plants) (Yearly gardening) |  |  |  |  |  |
| Nature-based adventure activities (such as surfing, climbing, hiking or camping in wilderness areas) (Yearly adventure) |  |  |  |  |  |

End of Block: Yearly nature contact

Start of Block: Weekly nature contact

Text In the above questions, you considered your participation in the last 12 months, in the following questions, we would like you to consider your participation in a typical week.

|  |  |
| --- | --- |

Weekly contact On how many days in a typical week do you participate in **ANY type of nature-based recreation and leisure activities**? *[For example, if you typically go to the beach 2 days in a week and garden on 2 different days out of that same week, your answer would be 4 days.]*

- None (never participate) (0)
- 1 day (1)
- 2 days (2)
- 3 days (3)
- 4 days (4)
- 5 days (5)
- 6 days (6)
- Every day (7)

| Page Break |  |
| --- | --- |

|  |  |
| --- | --- |

Weekly activities Next, tell us about your participation in specific types of nature-based recreation and leisure activities. On how many days in a typical week do you participate in the following activities?

|  | None (never participate) (0) | 1 day (1) | 2 days (2) | 3 days (3) | 4 days (4) | 5 days (5) | 6 days (6) | Every day (7) |
| --- | --- | --- | --- | --- | --- | --- | --- | --- |
| Activities in forested areas (such as parks or any other areas with many trees) (Weekly forest) |  |  |  |  |  |  |  |  |
| Activities related to gardening (such as planting and caring for plants) (Weekly gardening) |  |  |  |  |  |  |  |  |
| Nature-based adventure activities (such as surfing, climbing, hiking or camping in wilderness areas) (Weekly adventure) |  |  |  |  |  |  |  |  |

End of Block: Weekly nature contact

|  |  |
| --- | --- |

Attention When you participate in **ANY type of nature-based recreation and leisure activities**, which of the following best describes **your interaction with nature** (or natural elements such as plants and wildlife)?

- I **never** really pay attention to the natural world around me (0)
- I **rarely** pay attention to the natural world around me (1)
- I **sometimes** pay attention to the natural world around me (2)
- I **often** pay attention to the natural world around me (3)
- I **almost always** pay attention to the natural world around me (4)

|  |  |
| --- | --- |

Social When you participate in **ANY type of nature-based recreation and leisure activities** (that includes any of the activities listed above such as activities in forested areas, gardening, and adventure activities), which of the following best describes **who you participate with**?

- I **almost always** participate on my own (0)
- I **usually** participate on my own, but **sometimes** participate with other people (1)
- I participate on my own **about half the time** and participate with other people **about half the time** (2)
- I **usually** participate with other people, but **sometimes** participate on my own (3)
- I **almost always** participate with other people (4)

| Page Break |  |
| --- | --- |

|  |
| --- |

NatureConnection Finally, please indicate which picture best describes your relationship with the natural world. How interconnected are you with nature? (*Please click on ONE picture*.)


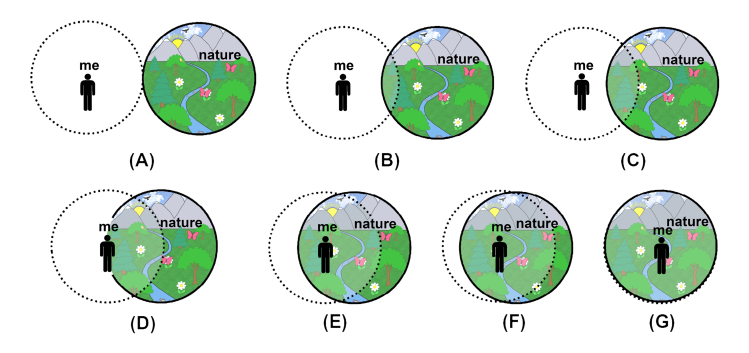


Text We are almost done. To finish, we would like to know a little bit more about you.

|  |  |
| --- | --- |

Age How old are you?

▼ younger than 18 (0) ... 100 (100)

|  |  |
| --- | --- |

Gender Which gender identity do you identify most strongly with?

- Man (0)
- Woman (1)
- Gender variant/Non-conforming (2)
- Not listed (3)

|  |  |
| --- | --- |

Education What is your highest level of **completed** education?

- Secondary (High) school (2)
- Vocational school (3)
- College or university (Bachelors or equivalent) (4)
- Graduate (Masters or equivalent) (5)
- Doctoral degree (6)

|  |  |
| --- | --- |

Income Please rate ***your family* income** relative to other people in the country where you live.

- Well below average (1)
- Slightly below average (2)
- Average (3)
- Slightly above average (4)
- Well above average (5)

| Page Break |  |
| --- | --- |

|  |  |
| --- | --- |

Ethnicity Which of the following best describes your racial/ethnic background? (Select ALL that apply.)

- American Indian or Alaska Native (1)
- Asian (2)
- Black or African American (3)
- Hispanic/Latino (4)
- Middle Eastern or North African (5)
- Mixed Race (6)
- Native Hawaiian or Pacific Islander (7)
- White (8)
- Other (please specify) (9) __________________________________________________

|  |  |
| --- | --- |

SkinColor
Please use the scale below the image to identify the color closest to your own skin color.

- Albino 0 (0)
- 1 (1)
-
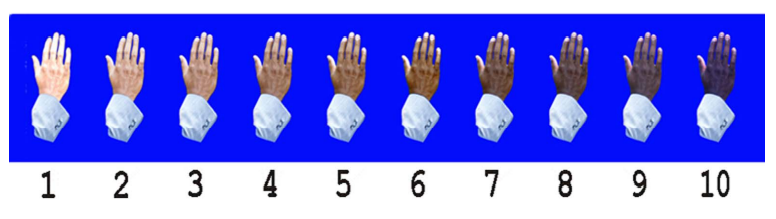
2 (2)
- 3 (3)
- 4 (4)
- 5 (5)
- 6 (6)
- 7 (7)
- 8 (8)
- 9 (9)
- 10 (10)

|  |  |
| --- | --- |

Urbanicity Would you describe the area where you live as urban or rural?

- Urban (1)
- Rural (2)
- Not sure (3)

**SPANISH VERSION**

**Encuesta sobre Naturaleza y Salud Mental**
Querido/a Participante,

Bienvenido/a a este estudio.

**Formulario de consentimiento informado**Te invitamos a participar de forma voluntaria en la investigación titulada Actividades en contacto con la naturaleza y síntomas de depresión en adultos. El objetivo es conocer la frecuencia de participación en actividades en contacto con la naturaleza y la ocurrencia de síntomas depresivos.

Para explorar esta relación te pedimos que, por favor, respondas al cuestionario que aparece a continuación con preguntas sobre actividades en contacto con la naturaleza y algunos síntomas de depresión. Se tarda unos 5-10 minutos.

Toda la información recopilada se utilizará exclusivamente con fines de investigación y se garantiza el anonimato. Para evitar que su identidad sea revelada, solo los investigadores responsables de la investigación tendrán acceso a la información que permita su identificación, como su dirección de correo electrónico. Puede dejar de completar el cuestionario en cualquier momento, sin penalización alguna, incluso después de aceptar participar. Su participación no supondrá ningún coste económico. Si tiene alguna pérdida que justifique daños y reparaciones, tendrá derecho a reclamar una indemnización. Si hay algún coste, se le reembolsará.

Puede ponerse en contacto con los investigadores principales de este estudio si necesita cualquier aclaración o ayuda, de forma gratuita y durante el tiempo que sea necesario. Después de rellenar el cuestionario recibirá un informe individual con su información y los resultados. Si acepta participar en este estudio, los datos registrados se utilizarán únicamente para la docencia y la investigación. Además, se puede descargar una copia de este formulario firmada por el investigador principal en el siguiente enlace https://osf.io/tk8wx.
   Investigador principal Brasil Claudio Damião Rosa (Ph.D. student)
 cdrosa@uesc.br
 55 73 991123063
 State University of Santa Cruz Investigadora principal España Dr. Silvia Collado (Profesora Titular de Universidad) scollado@unizar.es Universidad de Zaragoza
 **Por favor, haga click en el mensaje de abajo si acepta participar en el estudio**

- Entiendo de qué se trata la investigación y acepto participar (1)

|  |  |
| --- | --- |

Country ¿En qué país vives?

- Brasil (1)
- España (2)
- Estados Unidos de América (3)
- Otros (Por favor especifica): (4) __________________________________________________

Text En esta sección encontrarás algunas preguntas relacionadas con cómo te has sentido en las últimas 2 semanas.

|  |  |
| --- | --- |

WHO-5 Por favor, indica para cada una de las cinco afirmaciones cuál define mejor cómo te has sentido durante las últimas dos semanas

 En las últimas dos semanas:

|  | Nunca (0) | De vez en cuando (6) | Menos de la mitad del tiempo (1) | Más de la mitad del tiempo (2) | La mayor parte del tiempo (3) | Todo el tiempo (5) |
| --- | --- | --- | --- | --- | --- | --- |
| Me he sentido alegre y de buen humor (Cheerful) |  |  |  |  |  |  |
| Me he sentido tranquilo y relajado (Calm) |  |  |  |  |  |  |
| Me he sentido activo y enérgico (Active) |  |  |  |  |  |  |
| Me he despertado fresco y descansado (Rested) |  |  |  |  |  |  |
| Mi vida cotidiana ha estado llena de cosas que me interesan (Interested) |  |  |  |  |  |  |

| Page Break |  |
| --- | --- |

|  |  |
| --- | --- |

Symptoms1 Durante las últimas 2 semanas ¿con qué frecuencia te han molestado alguno de los siguientes problemas?

|  | Nunca (0) | Varios dias (1) | Más de la mitad de los dias (2) | Casi cada dia (3) |
| --- | --- | --- | --- | --- |
| Poco interés o alegría por hacer cosas (Pleasure) |  |  |  |  |
| Sensación de estar decaído/a, deprimido/a o desesperanzado/a (Depressed) |  |  |  |  |
| Problemas para quedarse dormido/a, para seguir durmiendo o dormir demasiado (Sleep) |  |  |  |  |
| Sensación de cansancio o de tener poca energía (Tired) |  |  |  |  |
| Poco apetito o comer demasiado (Appetite) |  |  |  |  |

|  |  |
| --- | --- |

Symptoms2 Durante las últimas 2 semanas ¿con qué frecuencia te han molestado alguno de los siguientes problemas?

|  | Nunca (0) | Varios dias (1) | Más de la mitad de los dias (2) | Casi cada dia (3) |
| --- | --- | --- | --- | --- |
| Sentirse mal consigo mismo/a; sentir que es un/a fracasado/a o que ha decepcionado a su familia o a sí mismo/a (Failure) |  |  |  |  |
| Problemas para concentrarse en algo, como leer el periódico o ver la televisión (Symptoms2_10) |  |  |  |  |
| Moverse o hablar tan despacio que los demás pueden haberlo notado. O lo contrario: estar tan inquieto/a o agitado/a que se ha estado moviendo de un lado a otro más de lo habitual (Symptoms2_11) |  |  |  |  |
| Pensamientos de que estaría mejor muerto/a o de querer hacerse daño de algún modo (Concentration) |  |  |  |  |

|  |  |
| --- | --- |

Difficulty Si has marcado alguno de los problemas de este cuestionario, ¿hasta qué punto estos problemas te han generado dificultades para hacer tu trabajo, ocuparte de la casa o relacionarte con los demás?

- No marqué ningún problema (99)
- Ninguna dificultad (1)
- Algunas dificultades (2)
- Muchas dificultades (3)
- Muchísimas dificultades (99)

| Page Break |  |
| --- | --- |

|  |  |
| --- | --- |

Treatment En caso de usar alguno, ¿cuál de los siguientes tratamientos estás usando actualmente para mejorar tu salud mental? (Marca todas las opciones que correspondan).

- Sin tratamiento (0)
- Psicoterapia y/o asesoramiento (1)
- Medicamento(s) prescritos por el médico (2)
- Otro (indícalo): (3) __________________________________________________

Text En esta sección encontrarás una serie de preguntas sobre tu participación en actividades de ocio en la naturaleza. Estamos interesados en diferentes tipos de actividades en la naturaleza y en saber con qué frecuencia las haces así que, por favor, lee atentamente cada una de las preguntas.

 Al contestar estas preguntas ten en cuenta que con **actividades de ocio en la naturaleza** nos referimos a actividades en contacto con la naturaleza que eliges hacer durante tu tiempo libre. Por ejemplo, pasar tiempo en un parque, senderismo, leer en la naturaleza, nadar en un lago o en el mar, o jardinería.

|  |  |
| --- | --- |

Yearly contact Primero, cuéntanos en qué medida participas de forma general en actividades de ocio en la naturaleza.
En los últimos 12 meses, ¿cuál de las siguientes afirmaciones describe mejor tu grado de participación en **CUALQUIER tipo de actividad de ocio en la naturaleza**?

- Nunca he participado (0)
- Rara vez he participado (pocas veces al año) (1)
- He participado a veces (aproximadamente una vez al mes) (2)
- He participado de manera habitual (varias veces al mes) (3)
- He participado casi siempre (prácticamente todas las semanas) (4)

| Page Break |  |
| --- | --- |

|  |  |
| --- | --- |

Yearly activities Ahora, cuéntanos acerca de tu participación en actividades específicas de ocio en la naturaleza. En los últimos 12 meses, ¿en qué medida has participado en las siguientes actividades?

|  | Nunca he participado (0) | Rara vez he participado (pocas veces al año) (1) | He participado a veces (aproximadamente una vez al mes) (2) | He participado de manera habitual (varias veces al mes) (3) | He participado casi siempre (prácticamente todas las semanas) (4) |
| --- | --- | --- | --- | --- | --- |
| Actividades en áreas boscosas (tales como parques o cualquier otra área con muchos árboles) (Yearly forest) |  |  |  |  |  |
| Actividades relacionas con la jardinería (tales como plantar o cuidar plantas) (Yearly gardening) |  |  |  |  |  |
| Actividades de aventura en la naturaleza (tales como surfear, escalar, senderismo o acampar en áreas silvestres) (Yearly adventure) |  |  |  |  |  |

Text En las preguntas anteriores, te hemos preguntado sobre tu participación en actividades en la naturaleza en los últimos 12 meses. Ahora nos gustaría que pensases en tu participación en lo que para ti es una semana típica.

|  |  |
| --- | --- |

Weekly contact En una semana típica, ¿cuántos días participas en **CUALQUIER tipo de actividad de ocio en la naturaleza**  *[Por ejemplo, si normalmente vas a la playa 2 veces a la semana y practicas la jardinería otros 2 días de la misma semana, tu respuesta sería 4 días.]*

- Ninguno (nunca participo) (0)
- 1 día (1)
- 2 días (2)
- 3 días (3)
- 4 días (4)
- 5 días (5)
- 6 días (6)
- Todos los días (7)

| Page Break |  |
| --- | --- |

|  |  |
| --- | --- |

Weekly activities Ahora, cuéntanos acerca de tu participación en **actividades específicas de ocio en la naturaleza**. En una semana típica, ¿cuántos días a la semana participas en las siguientes actividades?

|  | Ninguno (nunca participo) (0) | 1 día (1) | 2 días (2) | 3 días (3) | 4 días (4) | 5 días (5) | 6 días (6) | Todos los días (7) |
| --- | --- | --- | --- | --- | --- | --- | --- | --- |
| Actividades en áreas boscosas (tales como parques o cualquier otra área con muchos árboles) (Weekly forest) |  |  |  |  |  |  |  |  |
| Actividades relacionas con la jardinería (tales como plantar plantas o cuidar plantas) (Weekly gardening) |  |  |  |  |  |  |  |  |
| Actividades de aventura en la naturaleza (tales como surfear, escalar, senderismo o acampar en áreas silvestres) (Weekly adventure) |  |  |  |  |  |  |  |  |

|  |  |
| --- | --- |

Attention Cuando participas en **CUALQUIER tipo de actividades de ocio en la naturaleza**, ¿cuál de las siguientes afirmaciones describe mejor tu interacción con la naturaleza (o con los elementos naturales tales como las plantas y la fauna silvestre)?

- Nunca presto atención al mundo natural que me rodea (0)
- Rara vez presto atención al mundo natural que me rodea (1)
- A veces presto atención al mundo natural que me rodea (2)
- Normalmente presto atención al mundo natural que me rodea (3)
- Prácticamente siempre presto atención al mundo natural que me rodea (4)

|  |  |
| --- | --- |

Social Cuando participas en **CUALQUIER tipo de actividad recreativa y de ocio relacionada con la naturaleza** (que incluye todas las actividades listadas anteriormente tales como actividades en áreas boscosas, jardinería y actividades de aventura en la naturaleza), ¿cuál de las siguientes afirmaciones describe mejor **con quién participas?**

- Casi siempre participo solo/a (0)
- Normalmente participo solo/a, pero a veces con otras personas (1)
- Participo solo/a la mitad de las veces y con otras personas la otra mitad (2)
- Normalmente participo con otras personas, pero a veces participo solo/a (3)
- Casi siempre participo con otras personas (4)

| Page Break |  |
| --- | --- |

|  |
| --- |

NatureConnection Por favor, indica la figura que mejor represente tu relación con la naturaleza. ¿Cómo de conectado/a te sientes con la naturaleza? (*Por favor, haz clic en UNA imagen*.)


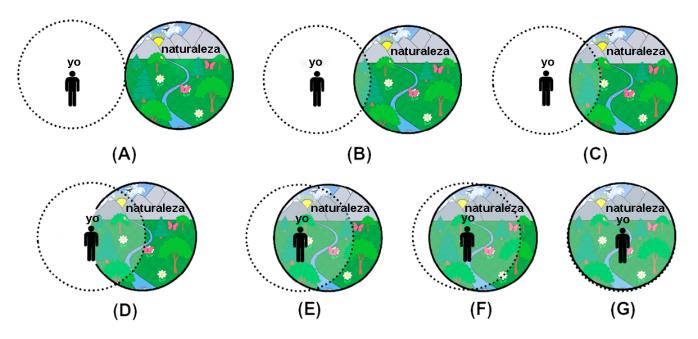


Text Ya casi hemos acabado. Para terminar, nos gustaría saber un poco más sobre ti.

|  |  |
| --- | --- |

Age ¿Cuántos años tienes?

▼ Menos de 18 (0) ... 100 (100)

|  |  |
| --- | --- |

Gender ¿Con qué género te identificas más?

- Masculino (0)
- Femenino (1)
- Género binario (2)
- Ninguno de los anteriores (3)

|  |  |
| --- | --- |

Education ¿Cuál es tu nivel de estudios? (marca el nivel de estudios terminados más alto)

- Secundaria y/o bachillerato (2)
- Formación profesional (3)
- Grado universitario (4)
- Máster (5)
- Doctorado (6)

|  |  |
| --- | --- |

Income Por favor, indica los **ingresos medios familiare**s que entran en tu casa en comparación con los ingresos de otras familias del país donde vives:

- Bastante por debajo de la media (1)
- Un poco por debajo de la media (2)
- En la media (3)
- Un poco por encima de la media (4)
- Bastante por encima de la media (5)

| Page Break |  |
| --- | --- |

|  |  |
| --- | --- |

Ethnicity ¿Cuál de las siguientes opciones te describe mejor? (Puedes marcar más de una)

- Blanco (1)
- Latino (2)
- Negro (3)
- Biracial (4)
- Asiatico (5)
- Otro (Por favor especifica) (9) __________________________________________________

|  |  |
| --- | --- |

SkinColor
Por favor, utiliza la escala de abajo para marcar el color que más se parece a tu color de piel.

- Albino 0 (0)
- 1 (1)
- 2 (2)
-
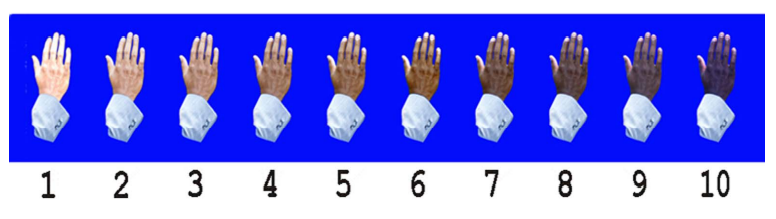
3 (3)
- 4 (4)
- 5 (5)
- 6 (6)
- 7 (7)
- 8 (8)
- 9 (9)
- 10 (10)

|  |  |
| --- | --- |

Urbanicity ¿Describirías la zona donde vives como urbana o rural?

- Urbana (1)
- Rural (2)
- No estoy seguro/a (3)

|  |
| --- |

Email Muchas gracias por tu ayuda. Nos encantaría poder contactarte en unas semanas con algunas preguntas de seguimiento. Prometemos no robarte mucho tiempo. Si estás de acuerdo, por favor, escribe tu dirección de correo electrónico.

________________________________________________________________

**PORTUGUESE-BR VERSION**

**Pesquisa sobre Natureza e Saúde Mental**

 Prezado(a) Participante,

 Bem-vindo(a) a este estudo.

 **Termo de Consentimento Livre e Esclarecido**

 Te convidamos a participar, como voluntário(a), em uma pesquisa que visa conhecer a frequência de participação em atividades em contacto com a natureza e a ocorrência de sintomas depressivos nas pessoas. Esta pesquisa intitula-se: Atividades em contato com a natureza e sintomas da depressão em adultos. Sua participação neste estudo aumentará nossa compreensão das possíveis conexões entre o tempo passado na natureza e os sentimentos/emoções das pessoas.

 Para explorar essas conexões, você responderá a um questionário nas páginas a seguir com perguntas sobre atividades em contato com a natureza e alguns sintomas da depressão. **O preenchimento do questionário deve levar de 5 a 10 minutos.**

 Todas as informações coletadas serão utilizadas exclusivamente para fins de pesquisa e o anonimato será garantido. Para evitar que sua identidade seja revelada, apenas os pesquisadores responsáveis terão acesso às informações que permitem sua identificação, como seu endereço de e-mail. Além disso, você pode sentir algum distúrbio psicológico, como cansaço, ao responder o questionário. Você pode interromper o preenchimento do questionário a qualquer momento e por qualquer motivo, sem qualquer penalidade, mesmo após concordar em participar. Sua participação não acarretará nenhum custo financeiro. Se tiver algum prejuízo que justifique danos e reparações, terá o direito de reclamar uma indenização. Se houver algum custo, você será reembolsado.

 Você pode entrar em contato com o pesquisador principal deste estudo se precisar de qualquer esclarecimento ou assistência, gratuitamente e pelo tempo que for necessário. Após participar da pesquisa, você receberá um relatório individual com suas informações e os resultados. Se você concordar em participar deste estudo, os dados registrados e algumas informações como dados socio-demográficos (sexo, idade e escolaridade) serão utilizados exclusivamente para ensino e pesquisa. Adicionalmente, uma cópia deste formulário, assinada pelo pesquisador responsável, pode ser baixada no seguinte link: https://osf.io/ntsx4/files/osfstorage/64235e2c3ed7dd29f0ecfa3f
   **Pesquisador principal** Claudio Damião Rosa (Doutorando)
 cdrosa@uesc.br
 55 73 991123063
 Universidade Estadual de Santa Cruz (UESC)


 **Por favor, clique na afirmativa abaixo se você concorda em participar do estudo.**

- Entendo do que se trata a pesquisa e concordo em participar (1)

|  |  |
| --- | --- |

Age Qual a sua idade?

▼ Menos de 18 (0) ... 100 (100)

|  |  |
| --- | --- |

Uni Você é um(a) estudante da Universidade Estadual de Santa Cruz (UESC)?

- Sim (1)
- Não (0)

|  |  |
| --- | --- |

Country Em que país você mora atualmente?

- Brasil (1)
- Espanha (2)
- Estados Unidos (3)
- Outro (por favor, especifique): (4) __________________________________________________

Text Nesta seção, você encontrará algumas perguntas relacionadas a como você tem se sentido nas últimas 2 semanas.

|  |  |
| --- | --- |

WHO-5 Por favor, indique para cada uma das 5 afirmações qual se aproxima mais de como você tem se sentido nas últimas 2 semanas.

Durante as últimas duas semanas:

|  | Nunca (0) | Algumas vezes (1) | Menos da metade do tempo (2) | Mais da metade do tempo (3) | A maior parte do tempo (4) | Todo o tempo (5) |
| --- | --- | --- | --- | --- | --- | --- |
| Senti-me alegre e bem disposto(a) (Cheerful) |  |  |  |  |  |  |
| Senti-me calmo(a) e tranquilo(a) (Calm) |  |  |  |  |  |  |
| Senti-me ativo(a) e enérgico(a) (Active) |  |  |  |  |  |  |
| Acordei a sentir-me revigorado(a) e descansado(a) (Rested) |  |  |  |  |  |  |
| O meu dia-a-dia tem sido preenchido com coisas que me interessam (Interested) |  |  |  |  |  |  |

| Page Break |  |
| --- | --- |

|  |  |
| --- | --- |

Symptoms1 Durante as últimas 2 semanas, com que frequência você foi incomodado(a) por qualquer um dos problemas abaixo?

|  | Nenhum dia (0) | Menos de uma semana (1) | Uma semana ou mais (2) | Quase todos os dias (3) |
| --- | --- | --- | --- | --- |
| Pouco interesse ou pouco prazer em fazer as coisas (Pleasure) |  |  |  |  |
| Se sentir “para baixo”, deprimido/a ou sem perspectiva (Depressed) |  |  |  |  |
| Dificuldade para pegar no sono ou permanecer dormindo ou dormir mais do que de costume (Sleep) |  |  |  |  |
| Se sentir cansado/a ou com pouca energia (Tired) |  |  |  |  |
| Falta de apetite ou comendo demais (Appetite) |  |  |  |  |

|  |  |
| --- | --- |

Symptoms2 Durante as últimas 2 semanas, com que frequência você foi incomodado(a) por qualquer um dos problemas abaixo?

|  | Nenhum dia (0) | Menos de uma semana (1) | Uma semana ou mais (2) | Quase todos os dias (3) |
| --- | --- | --- | --- | --- |
| Se sentir mal consigo mesmo(a) — ou achar que você é um fracasso ou que decepcionou sua família ou você mesmo(a) (Failure) |  |  |  |  |
| Dificuldade para se concentrar nas coisas, como ler o jornal ou ver televisão (Concentration) |  |  |  |  |
| Lentidão para se movimentar ou falar, a ponto das outras pessoas perceberem? Ou o oposto – estar tão agitado(a) ou irrequieto(a) que você fica andando de um lado para o outro muito mais do que de costume (Slow speech) |  |  |  |  |
| Pensar em se ferir de alguma maneira ou que seria melhor estar morto(a) (Thoughs) |  |  |  |  |

|  |  |
| --- | --- |

Difficulty Se você marcou qualquer um dos problemas acima, indique o grau de dificuldade que os mesmos lhe causaram para realizar seu trabalho, tomar conta das coisas em casa ou para se relacionar com as pessoas?

- Eu não marquei nenhum problema (99)
- Nenhuma dificuldade (1)
- Alguma dificuldade (2)
- Muita dificuldade (3)
- Extrema dificuldade (99)

| Page Break |  |
| --- | --- |

|  |  |
| --- | --- |

Treatment Qual dos seguintes tratamentos, se algum, você está usando atualmente para melhorar sua saúde mental? (Marque TODOS que se aplicam)

- Sem tratamento (0)
- Psicoterapia e/ou aconselhamento (1)
- Medicamento(s) prescrito(s) por um(a) médico(a) (2)
- Outro (por favor, especifique): (3) __________________________________________________

Text Aqui você encontrará algumas perguntas sobre sua participação em atividades de lazer na natureza. Estamos interessados em diferentes tipos de atividades na natureza e com que frequência você as realiza, portanto, leia cada pergunta com atenção.

 Ao responder a estas questões considere que as atividades de lazer na natureza são **atividades em contato com a natureza que você escolhe fazer no seu tempo livre**. Exemplos dessas atividades são visitas a parques, caminhar e fazer trilhas, leitura na natureza, natação em um lago ou oceano e jardinagem.

|  |  |
| --- | --- |

Yearly contact Primeiro, conte-nos sobre sua participação geral em atividades de lazer na natureza.

 Qual das seguintes opções melhor descreve sua participação em **QUALQUER tipo de atividade de lazer na natureza**, nos últimos 12 meses

- Eu nunca participei (0)
- Participei raramente (algumas vezes por ano) (1)
- Participei às vezes (cerca de uma vez por mês) (2)
- Participei frequentemente (várias vezes por mês) (3)
- Participei muito frequentemente (praticamente todas as semanas) (4)

| Page Break |  |
| --- | --- |

|  |  |
| --- | --- |

Yearly activities Em seguida, conte-nos sobre sua participação em tipos específicos de atividades de lazer na natureza.

Nos últimos 12 meses, com que frequência você se envolveu nas seguintes atividades?

|  | Eu nunca participei (0) | Participei raramente (algumas vezes por ano) (1) | Participei às vezes (cerca de uma vez por mês) (2) | Participei frequentemente (várias vezes por mês) (3) | Participei muito frequentemente (praticamente todas as semanas) (4) |
| --- | --- | --- | --- | --- | --- |
| Atividades em áreas florestadas (como espaços naturais protegidos ou quaisquer outras áreas com muitas árvores) (Yearly forest) |  |  |  |  |  |
| Atividades relacionadas à jardinagem (como plantar e cuidar de plantas) (Yearly gardening) |  |  |  |  |  |
| Atividades de aventura na natureza (como surf, escalada, trilhas a pé ou acampamento em áreas naturais pouco visitadas) (Yearly adventure) |  |  |  |  |  |

Text Nas perguntas acima, você considerou sua participação nos últimos 12 meses. Nas perguntas a seguir, gostaríamos que você considerasse sua participação em uma semana comum.

|  |  |
| --- | --- |

Weekly contact Em quantos dias de uma semana comum você participa de **QUALQUER tipo de atividades de lazer na natureza**? [Por exemplo, se você costuma ir à praia 2 dias na semana e praticar jardinagem em 2 dias diferentes da mesma semana, sua resposta seria 4 dias]

- Nenhum (nunca participo) (0)
- 1 dia (1)
- 2 dias (2)
- 3 dias (3)
- 4 dias (4)
- 5 dias (5)
- 6 dias (6)
- Todos os dias (7)

| Page Break |  |
| --- | --- |

|  |  |
| --- | --- |

Weekly activities Em seguida, conte-nos sobre sua participação em tipos específicos de atividades de lazer na natureza. Em quantos dias de uma semana comum você participa das seguintes atividades?

|  | Nenhum (nunca participo) (0) | 1 dia (1) | 2 dias (2) | 3 dias (3) | 4 dias (4) | 5 dias (5) | 6 dias (6) | Todos os dias (7) |
| --- | --- | --- | --- | --- | --- | --- | --- | --- |
| Atividades em áreas florestadas (como espaços naturais protegidos ou quaisquer outras áreas com muitas árvores) (Weekly forest) |  |  |  |  |  |  |  |  |
| Atividades relacionadas à jardinagem (como plantar e cuidar de plantas) (Weekly gardening) |  |  |  |  |  |  |  |  |
| Atividades de aventura na natureza (como surf, escalada, trilhas a pé ou acampamento em áreas naturais pouco visitadas) (Weekly adventure) |  |  |  |  |  |  |  |  |

|  |  |
| --- | --- |

Attention Quando você participa de **QUALQUER tipo de atividades de lazer na natureza**, qual das seguintes opções melhor descreve sua interação com a natureza (ou elementos naturais como plantas e vida selvagem)?

- Eu nunca presto atenção ao mundo natural ao meu redor (0)
- Raramente presto atenção ao mundo natural ao meu redor (1)
- Às vezes presto atenção ao mundo natural ao meu redor (2)
- Muitas vezes presto atenção ao mundo natural ao meu redor (3)
- Quase sempre presto atenção ao mundo natural ao meu redor (4)

|  |  |
| --- | --- |

Social Quando você participa de **QUALQUER tipo de atividades de lazer na natureza**, qual das opções a seguir melhor descreve com quem você participa?

- Quase sempre participo sozinho(a) (0)
- Geralmente participo sozinho(a), mas às vezes participo com outras pessoas (1)
- Eu participo sozinho(a) cerca de metade do tempo e participo com outras pessoas cerca de metade do tempo (2)
- Geralmente participo com outras pessoas, mas às vezes participo sozinho(a) (3)
- Quase sempre participo com outras pessoas (4)

| Page Break |  |
| --- | --- |

|  |
| --- |

NatureConnection Finalmente, por favor, indique qual imagem melhor descreve seu relacionamento com o mundo natural. Quão interconectado está você com a natureza?


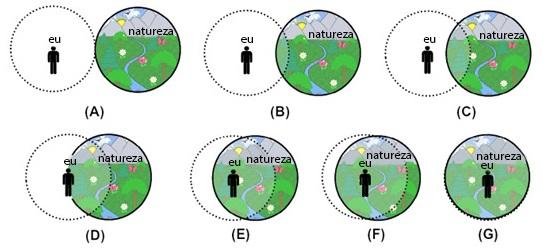


Text Estamos quase terminando. Para finalizar, gostaríamos de saber um pouco mais sobre você.

|  |  |
| --- | --- |

Gender Com qual identidade de gênero você se identifica mais fortemente?

- Homem (0)
- Mulher (1)
- Variação de gênero/não binário (2)
- Não listado (3)

|  |  |
| --- | --- |

Education Qual é o seu nível mais alto de **educação concluída**?

- Ensino médio (2)
- Ensino profissionalizante (3)
- Graduação (4)
- Mestrado (5)
- Doutorado (6)

|  |  |
| --- | --- |

Income Por favor, avalie sua **renda familiar** em relação a outras pessoas no país onde você mora?

- Bem abaixo da média (1)
- Um pouco abaixo da média (2)
- Na média (3)
- Um pouco acima da média (4)
- Bem acima da média (5)

| Page Break |  |
| --- | --- |

|  |  |
| --- | --- |

Ethnicity Qual das seguintes opções melhor descreve sua cor?

- Branca (1)
- Parda (2)
- Preta (3)
- Amarela (4)
- Outra (por favor, especifique) (9) __________________________________________________

|  |  |
| --- | --- |

SkinColor
Use a imagem abaixo para identificar a cor mais próxima da sua cor de pele 

- Albino 0 (0)
- 1 (1)
- 2 (2)
-
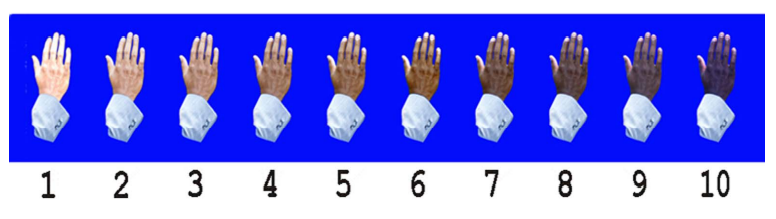
3 (3)
- 4 (4)
- 5 (5)
- 6 (6)
- 7 (7)
- 8 (8)
- 9 (9)
- 10 (10)

|  |  |
| --- | --- |

Urbanicity Você descreveria a área onde você mora como urbana ou rural?

- Urbana (1)
- Rural (2)
- Não tenho certeza (3)

|  |
| --- |

Email Muito obrigado por sua ajuda. Gostariamos de entrar em contato com você após algumas semanas com perguntas de acompanhamento. Prometemos não tomar muito do seu tempo. Se você concorda, digite seu endereço de e-mail.

________________________________________________________________

**REFERENCES**

Anderson, M. R., Salisbury, A. L., Uebelacker, L. A., Abrantes, A. M., & Battle, C. L. (2022). Stress, coping and silver linings: How depressed perinatal women experienced the COVID-19 pandemic. *Journal of Affective Disorders*, *298*, 329–336. https://doi.org/10.1016/j.jad.2021.10.116

Antonakis, J., Bendahan, S., Jacquart, P., & Lalive, R. (2010). On making causal claims: A review and recommendations. *The Leadership Quarterly*, *21*(6), 1086–1120. https://doi.org/10.1016/j.leaqua.2010.10.010

Astell-Burt, T., Hartig, T., Putra, I. G. N. E., Walsan, R., Dendup, T., & Feng, X. (2022). Green space and loneliness: A systematic review with theoretical and methodological guidance for future research. *Science of The Total Environment*, *847*(157521), 1–16. https://doi.org/10.1016/j.scitotenv.2022.157521

Barger, Torquati, Larson, Bartz, Johnson-Gaither, Gardner, Moody, Rosenberg, Schutte, Murray, & Schram. (2021). Measuring Green Space Effects on Attention and Stress in Children and Youth: A Scoping Review. *Children, Youth and Environments*, *31*(1), 1–54. https://doi.org/10.7721/chilyoutenvi.31.1.0001

Basu, M., DasGupta, R., Kumar, P., & Dhyani, S. (2021). Home gardens moderate the relationship between Covid-19-induced stay-at-home orders and mental distress: a case study with urban residents of India. *Environmental Research Communications*, *3*(105002), 1–13. https://doi.org/10.1088/2515-7620/ac2ab2

Beute, F., Davies, Z., de Vries, S., Glanville, J., Keune, H., Lammel, A., Marselle, M., O’Brien, L., Olszewska-Guizzo, A., Remmen, R., Russon, A., & Andreucci, M. B. (2021). *Types and characteristics of urban and peri-urban blue spaces having an impact on human mental health and wellbeing: A systematic review*. UK Centre for Ecology & Hydrology.

Braçe, O., Garrido-Cumbrera, M., Foley, R., Correa-Fernández, J., Suárez-Cáceres, G., & Lafortezza, R. (2020). Is a view of green spaces from home associated with a lower risk of anxiety and depression? *International Journal of Environmental Research and Public Health*, *17*(19), 1–9. https://doi.org/10.3390/ijerph17197014

Bressane, A., Negri, R. G., de Brito Junior, I., Medeiros, L. C. de C., Araújo, I. L. L., Silva, M. B., Galvão, A. L. dos S., & Rosa, G. C. S. da. (2022). Association between Contact with Nature and Anxiety, Stress and Depression Symptoms: A Primary Survey in Brazil. *Sustainability*, *14*(17), 1–10. https://doi.org/10.3390/su141710506

Britton, E., Kindermann, G., Domegan, C., & Carlin, C. (2020). Blue care: A systematic review of blue space interventions for health and wellbeing. *Health Promotion International*, *35*(1), 50–69. https://doi.org/10.1093/heapro/day103

Browning, M. H. E. M., Saeidi-Rizi, F., McAnirlin, O., Yoon, H., & Pei, Y. (2021). The role of methodological choices in the effects of experimental exposure to simulated natural landscapes on human health and cognitive performance: A systematic review. *Environment and Behavior*, *53*(7), 687–731. https://doi.org/10.1177/0013916520906481

Bu, F., Steptoe, A., Mak, H. W., & Fancourt, D. (2021). Time use and mental health in UK adults during an 11-week COVID-19 lockdown: a panel analysis. *The British Journal of Psychiatry*, *219*(4), 551–556. https://doi.org/10.1192/bjp.2021.44

Corley, J., Okely, J. A., Taylor, A. M., Page, D., Welstead, M., Skarabela, B., Redmond, P., Cox, S. R., & Russ, T. C. (2021). Home garden use during COVID-19: Associations with physical and mental wellbeing in older adults. *Journal of Environmental Psychology*, *73*, 1–8. https://doi.org/10.1016/j.jenvp.2020.101545

Coventry, P. A., Brown, J. E., Pervin, J., Brabyn, S., Pateman, R., Breedvelt, J., Gilbody, S., Stancliffe, R., McEachan, R., & White, P. L. (2021). Nature-based outdoor activities for mental and physical health: Systematic review and meta-analysis. *SSM - Population Health*, *16*(100934), 1–14. https://doi.org/10.1016/j.ssmph.2021.100934

Cox, D. T. C., Shanahan, D. F., Hudson, H. L., Fuller, R. A., Anderson, K., Hancock, S., & Gaston, K. J. (2017). Doses of nearby nature simultaneously associated with multiple health benefits. *International Journal of Environmental Research and Public Health*, *14*(172), 1–13. https://doi.org/10.3390/ijerph14020172

Cox, D. T. C., Shanahan, D. F., Hudson, H. L., Fuller, R. A., & Gaston, K. J. (2018). The impact of urbanisation on nature dose and the implications for human health. *Landscape and Urban Planning*, *179*, 72–80. https://doi.org/10.1016/j.landurbplan.2018.07.013

Das, A., & Gailey, S. (2022). Green exercise, mental health symptoms, and state lockdown policies: A longitudinal study. *Journal of Environmental Psychology*, *82*(101848), 1–8. https://doi.org/10.1016/j.jenvp.2022.101848

Davis, Z., Guhn, M., Jarvis, I., Jerrett, M., Nesbitt, L., Oberlander, T., Sbihi, H., Su, J., & van den Bosch, M. (2021). The association between natural environments and childhood mental health and development: A systematic review and assessment of different exposure measurements. *International Journal of Hygiene and Environmental Health*, *235*(113767), 1–13. https://doi.org/10.1016/j.ijheh.2021.113767

Frost, S., Kannis-Dymand, L., Schaffer, V., Millear, P., Allen, A., Stallman, H., Mason, J., Wood, A., & Atkinson-Nolte, J. (2022). Virtual immersion in nature and psychological well-being: A systematic literature review. *Journal of Environmental Psychology*, *80*(101765), 1–9. https://doi.org/10.1016/j.jenvp.2022.101765

Geneshka, M., Coventry, P., Cruz, J., & Gilbody, S. (2021). Relationship between Green and Blue Spaces with Mental and Physical Health: A Systematic Review of Longitudinal Observational Studies. *International Journal of Environmental Research and Public Health*, *18*(17), 1–29. https://doi.org/10.3390/ijerph18179010

Gubbels, J. S., Kremers, S. P. J., Droomers, M., Hoefnagels, C., Stronks, K., Hosman, C., & de Vries, S. (2016). The impact of greenery on physical activity and mental health of adolescent and adult residents of deprived neighborhoods: A longitudinal study. *Health & Place*, *40*, 153–160. https://doi.org/10.1016/j.healthplace.2016.06.002

Heo, S., Desai, M. U., Lowe, S. R., & Bell, M. L. (2021). Impact of Changed Use of Greenspace during COVID-19 Pandemic on Depression and Anxiety. *International Journal of Environmental Research and Public Health*, *18*(5842), 1–18. https://doi.org/10.3390/ijerph18115842

Higgins, J. P. T., Thomas, J., Chandler, J., Cumpston, M., Li, T., Page, M. J., & Welch, V. A. (Eds.). (2019). *Cochrane Handbook for Systematic Reviews of Interventions* (2nd ed.). John Wiley & Sons.

Howarth, M., Brettle, A., Hardman, M., & Maden, M. (2020). What is the evidence for the impact of gardens and gardening on health and well-being: A scoping review and evidence-based logic model to guide healthcare strategy decision making on the use of gardening approaches as a social prescription. *BMJ Open*, *10*(e036923), 1–16. https://doi.org/10.1136/bmjopen-2020-036923

Hubbard, G., Daas, C. den, Johnston, M., Murchie, P., Thompson, C. W., & Dixon, D. (2021). Are Rurality, Area Deprivation, Access to Outside Space, and Green Space Associated with Mental Health during the COVID-19 Pandemic? A Cross Sectional Study (CHARIS-E). *International Journal of Environmental Research and Public Health*, *18*(8), 1–17. https://doi.org/10.3390/ijerph18083869

Jones, R., Tarter, R., & Ross, A. M. (2021). Greenspace Interventions, Stress and Cortisol: A Scoping Review. *International Journal of Environmental Research and Public Health*, *18*(2802), 1–21. https://doi.org/10.3390/ijerph18062802

Kotera, Y., Lyons, M., Vione, K. C., & Norton, B. (2021). Effect of nature walks on depression and anxiety: A systematic review. *Sustainability*, *13*(7), 4015. https://doi.org/10.3390/su13074015

Labib, S. M., Lindley, S., & Huck, J. J. (2020). Spatial dimensions of the influence of urban green-blue spaces on human health: A systematic review. *Environmental Research*, *180*(108869), 1–22. https://doi.org/10.1016/j.envres.2019.108869

Li, H., Zhang, X., Bi, S., Cao, Y., & Zhang, G. (2022). Psychological benefits of green exercise in wild or urban greenspaces: A meta-analysis of controlled trials. *Urban Forestry & Urban Greening*, *68*(127458), 1–8. https://doi.org/10.1016/j.ufug.2022.127458

Li, H., Zhang, X., You, C., Chen, X., Cao, Y., & Zhang, G. (2021). Can Viewing Nature Through Windows Improve Isolated Living? A Pathway Analysis on Chinese Male Prisoners During the COVID-19 Epidemic. *Frontiers in Psychiatry*, *12*(720722), 1–10. https://doi.org/10.3389/fpsyt.2021.720722

Lõhmus, M., Stenfors, C. U. D., Lind, T., Lauber, A., & Georgelis, A. (2021). Mental health, greenness, and nature related behaviors in the adult population of Stockholm County during COVID-19-related restrictions. *International Journal of Environmental Research and Public Health*, *18*(3303), 1–21. https://doi.org/10.3390/ijerph18063303

Marini, S., Mauro, M., Grigoletto, A., Toselli, S., & Maietta Latessa, P. (2022). The Effect of Physical Activity Interventions Carried Out in Outdoor Natural Blue and Green Spaces on Health Outcomes: A Systematic Review. *International Journal of Environmental Research and Public Health*, *19*(12482), 1–15. https://doi.org/10.3390/ijerph191912482

Marques, P., Silva, A. S., Quaresma, Y., Manna, L. R., de Magalhães Neto, N., & Mazzoni, R. (2021). Home gardens can be more important than other urban green infrastructure for mental well-being during COVID-19 pandemics. *Urban Forestry & Urban Greening*, *64*(127268), 1–6. https://doi.org/10.1016/j.ufug.2021.127268

Marselle, M., Warber, S., & Irvine, K. (2019). Growing Resilience through Interaction with Nature: Can Group Walks in Nature Buffer the Effects of Stressful Life Events on Mental Health? *International Journal of Environmental Research and Public Health*, *16*(986). https://doi.org/10.3390/ijerph16060986

Nigg, C., Petersen, E., & MacIntyre, T. (2021). *Natural environments, psychosocial health, and health behaviors in a crisis – a scoping review of the literature in the COVID-19 context*. https://doi.org/10.31234/osf.io/a9unf

Olszewska-Guizzo, A., Fogel, A., Escoffier, N., & Ho, R. (2021). Effects of COVID-19-related stay-at-home order on neuropsychophysiological response to urban spaces: Beneficial role of exposure to nature? *Journal of Environmental Psychology*, *75*, 101590. https://doi.org/10.1016/j.jenvp.2021.101590

Oswald, T. K., Rumbold, A. R., Kedzior, S. G. E., & Moore, V. M. (2020). Psychological impacts of “screen time” and “green time” for children and adolescents: A systematic scoping review. *PLOS ONE*, *15*(e0237725), 1–52. https://doi.org/10.1371/journal.pone.0237725

Pouso, S., Borja, Á., Fleming, L. E., Gómez-Baggethun, E., White, M. P., & Uyarra, M. C. (2021). Contact with blue-green spaces during the COVID-19 pandemic lockdown beneficial for mental health. *Science of The Total Environment*, *756*(143984), 1–12. https://doi.org/10.1016/j.scitotenv.2020.143984

Quarta, S., Levante, A., García-Conesa, M.-T., Lecciso, F., Scoditti, E., Carluccio, M. A., Calabriso, N., Damiano, F., Santarpino, G., Verri, T., Pinto, P., Siculella, L., & Massaro, M. (2022). Assessment of subjective well-being in a cohort of university students and staff members: Association with physical activity and outdoor leisure time during the covid-19 pandemic. *International Journal of Environmental Research and Public Health*, *19*(8), 1–26. https://doi.org/10.3390/ijerph19084787

Reid, C. E., Rieves, E. S., & Carlson, K. (2022). Perceptions of green space usage, abundance, and quality of green space were associated with better mental health during the COVID-19 pandemic among residents of Denver. *PLOS ONE*, *17*(3), e0263779. https://doi.org/10.1371/journal.pone.0263779

Reklaitiene, R., Grazuleviciene, R., Dedele, A., Virviciute, D., Vensloviene, J., Tamosiunas, A., Baceviciene, M., Luksiene, D., Sapranaviciute-Zabazlajeva, L., Radisauskas, R., Bernotiene, G., Bobak, M., & Nieuwenhuijsen, M. J. (2014). The relationship of green space, depressive symptoms and perceived general health in urban population. *Scandinavian Journal of Public Health*, *42*(7), 669–676. https://doi.org/10.1177/1403494814544494

Ricciardi, E., Spano, G., Tinella, L., Lopez, A., Clemente, C., Bosco, A., & Caffò, A. O. (2023). Perceived social support mediates the relationship between use of greenspace and geriatric depression: A cross-sectional study in a sample of south-italian older adults. *International Journal of Environmental Research and Public Health*, *20*(5540), 1–11. https://doi.org/10.3390/ijerph20085540

Rosa, C. D., Larson, L. R., Collado, S., & Profice, C. C. (2021). Forest therapy can prevent and treat depression: Evidence from meta-analyses. *Urban Forestry & Urban Greening*, *57*, 126943. https://doi.org/10.1016/j.ufug.2020.126943

Samus, A., Freeman, C., Dickinson, K. J. M., & van Heezik, Y. (2022). Relationships between nature connectedness, biodiversity of private gardens, and mental well-being during the Covid-19 lockdown. *Urban Forestry & Urban Greening*, *69*(127519), 1–9. https://doi.org/10.1016/j.ufug.2022.127519

Shanahan, D. F., Bush, R., Gaston, K. J., Lin, B. B., Dean, J., Barber, E., & Fuller, R. A. (2016). Health benefits from nature experiences depend on dose. *Scientific Reports*, *6*(28551). https://doi.org/10.1038/srep28551

Shin, J. C., Parab, K. V., An, R., & Grigsby-Toussaint, D. S. (2020). Greenspace exposure and sleep: A systematic review. *Environmental Research*, *182*(109081). https://doi.org/10.1016/j.envres.2019.109081

Snell, T. L., Lam, J. C. S., Lau, W. W.-Y., Lee, I., Maloney, E. M., Mulholland, N., Wilson, L., & Wynne, L. J. (2016). Contact with nature in childhood and adult depression. *Children, Youth and Environments*, *26*(1), 111–124. https://doi.org/10.1353/cye.2016.0018

Sterne, J. A., Hernán, M. A., Reeves, B. C., Savović, J., Berkman, N. D., Viswanathan, M., Henry, D., Altman, D. G., Ansari, M. T., Boutron, I., Carpenter, J. R., Chan, A.-W., Churchill, R., Deeks, J. J., Hróbjartsson, A., Kirkham, J., Jüni, P., Loke, Y. K., Pigott, T. D., … Higgins, J. P. (2016). ROBINS-I: A tool for assessing risk of bias in non-randomised studies of interventions. *BMJ*, *i4919*. https://doi.org/10.1136/bmj.i4919

Sterne, J. A., Savović, J., Page, M. J., Elbers, R. G., Blencowe, N. S., Boutron, I., Cates, C. J., Cheng, H.-Y., Corbett, M. S., Eldridge, S. M., Emberson, J. R., Hernán, M. A., Hopewell, S., Hróbjartsson, A., Junqueira, D. R., Jüni, P., Kirkham, J. J., Lasserson, T., Li, T., … Higgins, J. P. T. (2019). RoB 2: A revised tool for assessing risk of bias in randomised trials. *BMJ*, l4898. https://doi.org/10.1136/bmj.l4898

Svensson, M., Brundin, L., Erhardt, S., Madaj, Z., Hållmarker, U., James, S., & Deierborg, T. (2019). Long distance ski racing is associated with lower long-term incidence of depression in a population based, large-scale study. *Psychiatry Research*, *281*(112546), 1–7. https://doi.org/10.1016/j.psychres.2019.112546

Tomasi, S., Di Nuovo, S., & Hidalgo, M. C. (2020). Environment and mental health: empirical study on the relationship between contact with nature and symptoms of anxiety and depression. *PsyEcology*, *11*(3), 319–341. https://doi.org/10.1080/21711976.2020.1778388

Torres, E. R., Sampselle, C. M., Ronis, D. L., Neighbors, H. W., & Gretebeck, K. A. (2016). Gardening/yard work and depressive symptoms in African Americans. *Archives of Psychiatric Nursing*, *30*(2), 155–161. https://doi.org/10.1016/j.apnu.2015.08.004

Trevino, J. E., Monsur, M., Lindquist, C. S., & Simpson, C. R. (2022). Student and nature interactions and their impact on mental health during the COVID-19 pandemic. *International Journal of Environmental Research and Public Health*, *19*(9), 1–17. https://doi.org/10.3390/ijerph19095030

van Lier, L. E., Utter, J., Denny, S., Lucassen, M., Dyson, B., & Clark, T. (2017). Home gardening and the health and well-being of adolescents. *Health Promotion Practice*, *18*(1), 34–43. https://doi.org/10.1177/1524839916673606

Vibholm, A. P., Christensen, J. R., & Pallesen, H. (2020). Nature-based rehabilitation for adults with acquired brain injury: a scoping review. *International Journal of Environmental Health Research*, *30*(6), 661–676. https://doi.org/10.1080/09603123.2019.1620183

von Elm, E., Altman, D. G., Egger, M., Pocock, S. J., Gøtzsche, P. C., & Vandenbroucke, J. P. (2007). The Strengthening the Reporting of Observational Studies in Epidemiology (STROBE) Statement: Guidelines for Reporting Observational Studies. *Annals of Internal Medicine*, *147*(8), 573. https://doi.org/10.7326/0003-4819-147-8-200710160-00010

Wilkie, S., & Davinson, N. (2021). Prevalence and effectiveness of nature-based interventions to impact adult health-related behaviours and outcomes: A scoping review. *Landscape and Urban Planning*, *214*(104166), 2–10. https://doi.org/10.1016/j.landurbplan.2021.104166

Williams, T., Barnwell, G. C., & Stein, D. J. (2020). *A Systematic Review of Randomised Controlled Trials on the Effectiveness of Ecotherapy Interventions for Treating Mental Disorders*. https://doi.org/https://doi.org/10.1101/2020.09.25.20201525

Wolsko, C., Lindberg, K., & Reese, R. (2019). Nature-Based Physical Recreation Leads to Psychological Well-Being: Evidence from Five Studies. *Ecopsychology*, eco.2018.0076. https://doi.org/10.1089/eco.2018.0076

Wood, C., Barron, D., & Smyth, N. (2019). The Current and Retrospective Intentional Nature Exposure Scales: Development and Factorial Validity. *International Journal of Environmental Research and Public Health*, *16*(4443), 1–18. https://doi.org/10.3390/ijerph16224443

Yao, W., Zhang, X., & Gong, Q. (2021). The effect of exposure to the natural environment on stress reduction: A meta-analysis. *Urban Forestry & Urban Greening*, *57*(126932), 1–12. https://doi.org/10.1016/j.ufug.2020.126932

Yen, H.-Y., Chiu, H.-L., & Huang, H.-Y. (2021). Green and blue physical activity for quality of life: A systematic review and meta-analysis of randomized control trials. *Landscape and Urban Planning*, *212*(104093). https://doi.org/10.1016/j.landurbplan.2021.104093

Yi, Y., Seo, E., & An, J. (2022). Does Forest Therapy Have Physio-Psychological Benefits? A Systematic Review and Meta-Analysis of Randomized Controlled Trials. *International Journal of Environmental Research and Public Health*, *19*(10512), 2–21. https://doi.org/10.3390/ijerph191710512

Zhang, J., Browning, M. H. E. M., Liu, J., Cheng, Y., Zhao, B., & Dadvand, P. (2023). Is indoor and outdoor greenery associated with fewer depressive symptoms during COVID-19 lockdowns? A mechanistic study in Shanghai, China. *Building and Environment*, *227*(109799), 1–10. https://doi.org/10.1016/j.buildenv.2022.109799

Zhang, J., Yu, Z., Zhao, B., Sun, R., & Vejre, H. (2020). Links between green space and public health: a bibliometric review of global research trends and future prospects from 1901 to 2019. *Environmental Research Letters*, *15*(063001), 1–18. https://doi.org/10.1088/1748-9326/ab7f64
